# D-Glucosamine supplementation extends life span of nematodes and of ageing mice

**DOI:** 10.1038/ncomms4563

**Published:** 2014-04-08

**Authors:** Sandra Weimer, Josephine Priebs, Doreen Kuhlow, Marco Groth, Steffen Priebe, Johannes Mansfeld, Troy L. Merry, Sébastien Dubuis, Beate Laube, Andreas F. Pfeiffer, Tim J. Schulz, Reinhard Guthke, Matthias Platzer, Nicola Zamboni, Kim Zarse, Michael Ristow

**Affiliations:** 1 Energy Metabolism Laboratory, ETH Zürich (Swiss Federal Institute of Technology Zurich), Zürich CH-8603, Switzerland; 2 German Institute of Human Nutrition Potsdam-Rehbrücke, D-14558 Nuthetal, Germany; 3 Department of Human Nutrition, Institute of Nutrition, University of Jena, D-07743 Jena, Germany; 4 Genome Analysis Group, Leibniz Institute for Age Research, Fritz-Lipmann-Institute, D-07745 Jena, Germany; 5 Systems Biology and Bioinformatics Group, Leibniz Institute for Natural Product Research and Infection Biology, Hans-Knöll-Institute, D-07745 Jena, Germany; 6 DFG Graduate School of Adaptive Stress Response #1715, D-07745 Jena, Germany; 7 Institute of Molecular Systems Biology, ETH Zürich (Swiss Federal Institute of Technology Zurich), Zürich CH-8093, Switzerland; 8 These authors contributed equally to the work

## Abstract

D-Glucosamine (GlcN) is a freely available and commonly used dietary supplement potentially promoting cartilage health in humans, which also acts as an inhibitor of glycolysis. Here we show that GlcN, independent of the hexosamine pathway, extends *Caenorhabditis elegans* life span by impairing glucose metabolism that activates AMP-activated protein kinase (AMPK/AAK-2) and increases mitochondrial biogenesis. Consistent with the concept of mitohormesis, GlcN promotes increased formation of mitochondrial reactive oxygen species (ROS) culminating in increased expression of the nematodal *amino acid-transporter 1* (*aat-1*) gene. Ameliorating mitochondrial ROS formation or impairment of *aat-1*-expression abolishes GlcN-mediated life span extension in an NRF2/SKN-1-dependent fashion. Unlike other calorie restriction mimetics, such as 2-deoxyglucose, GlcN extends life span of ageing C57BL/6 mice, which show an induction of mitochondrial biogenesis, lowered blood glucose levels, enhanced expression of several murine amino-acid transporters, as well as increased amino-acid catabolism. Taken together, we provide evidence that GlcN extends life span in evolutionary distinct species by mimicking a low-carbohydrate diet.

## Abstract (web summary)

D-Glucosamine is a dietary supplement widely used for the treatment of osteoarthritis. Here Weimer *et al.* show that D-glucosamine extends the life span of *Caenorhabditis elegan*s and of mice by mimicking the molecular effects of a diet low in carbohydrates.

## Graphical abstract

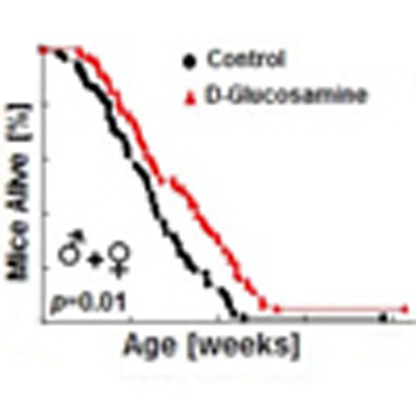

D-Glucosamine (GlcN) (2-amino-2-deoxy-D-glucose, CAS 3416-24-8) is being widely used to prevent and treat osteoarthritis in humans and, according to a number of clinical studies^1^,^2^, may be effective in this regard. However, mounting evidence suggests that GlcN may be ineffective in ameliorating symptoms and parameters of osteoarthritis^3^. Nevertheless, GlcN has been in long-term use in humans for several decades and induces no relevant side effects aside from occasional allergic reactions.

GlcN in its endogenously phosphorylated form, GlcN-6-phosphate, is a well-established inhibitor of both hexokinase^4^ (EC enzyme number 2.7.1.1) and, in particular, its predominant liver-specific functional isoform, glucokinase^5^ (EC 2.7.1.2), thereby reflecting the initial step within the enzymatic breakdown of glucose to form pyruvate and ATP, which is called glycolysis. Accordingly, short-term administration of high-dose GlcN to model systems^6^,^7^,^8^ or humans^9^,^10^ acutely impairs glucose metabolism that resembles some of the metabolic features of diabetes mellitus. By contrast, chronic GlcN intake has no detectable influence^11^, or even blood glucose-lowering^12^ effects in humans.

Long-term inhibition of glycolysis, by either applying RNA interference (RNAi) to impair expression of glycolytic enzymes^13^,^14^,^15^, with the application of 2-deoxy-D-glucose (DOG)^15^, or by impeding insulin/IGF1 receptor signalling^16^ uniformly extends the life span of *C. elegans*, whereas increased glucose availability reduces nematodal life span^15^,^17^,^18^. As none of these aforementioned interventions are readily available for use in humans to extend life span, and particularly owing to the fact that DOG unexpectedly shortens life span of rodents^19^,^20^, we have now tested whether GlcN could promote healthspan in *C. elegans* and rodents.

We here find that GlcN inhibits glycolysis to cause an energy deficit that induces mitochondrial biogenesis and alternate fuel use, namely amino-acid oxidation. This is paralleled by an extension of life span in both *C. elegans* and ageing mice, the latter also showing improved glucose metabolism. These findings implicate that GlcN supplementation may be a versatile approach to delay ageing in humans.

## Results

### GlcN impairs glycolysis and extends life span in nematodes

**Figure 1:**
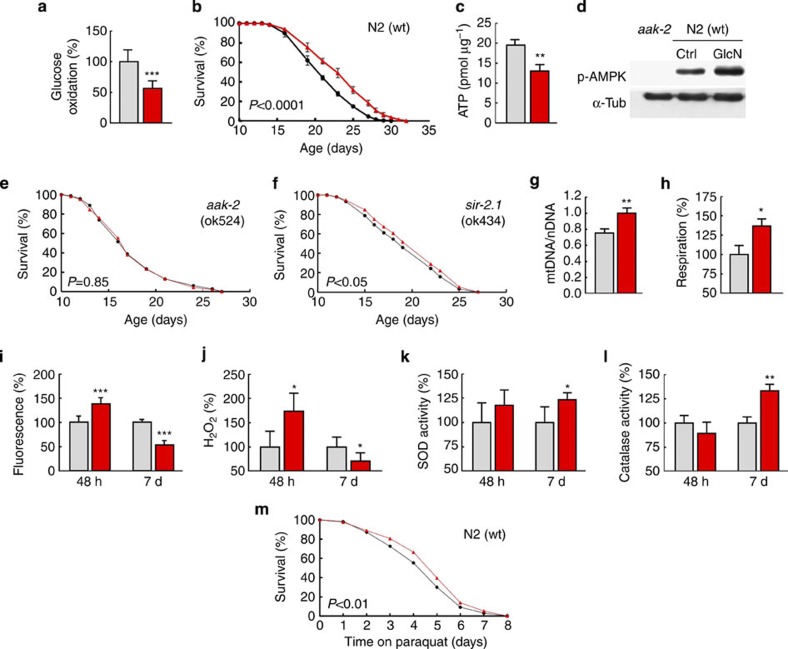
GlcN induces mitochondrial metabolism and extends *C. elegans* life span. (**a**) Glucose oxidation rates in control wild-type (wt) nematodes (grey) and wild-type nematodes exposed to GlcN (red) (*P*<0.001, Student’s *t*-test, *n*=6); colour coding applies to all subsequent panels and figures. (**b**) Life expectancy of untreated wild-type-nematodes and GlcN-treated *C. elegans* (*P*<0.0001, log-rank test, *n*=3). (**c**) ATP content at different time points in GlcN- and untreated nematodes (*P*<0.01, Student’s *t*-test, *n*=3). (**d**) Representative western blot of whole-worm lysates in the presence and absence of GlcN in wild-type worms, as well as untreated AAK-2-deficient worms. (**e**) Life span assay on AAK-2-deficient nematodes in the presence and absence of GlcN (*P*=0.85, log-rank test, *n*=3). (**f**) Life span assay on SIR-2.1-deficient nematodes (*P*<0.05, log-rank test, *n*=3). (**g**) mtDNA content normalized to nuclear DNA content in whole worms in the presence and absence of GlcN (*P*<0.01, Student’s *t*-test, *n*=3). (**h**) Relative respiration rates of whole worms (*P*<0.05, Student’s *t*-test, *n*=3), and (**i**) relative MitoTracker Red CM-H_2_X fluorescence of whole worms in the presence and absence of GlcN at different time points (*P*<0.001, Student’s *t*-test, *n*=3). (**j**) Relative Amplex Red fluorescence in suspensions of alive nematodes (*P*<0.05, Student’s *t*-test, *n*=3). (**k**) Relative superoxide dismutase activities (*P*<0.05, Student’s *t*-test, *n*=3). (**l**) Relative catalase activities (*P*<0.01, Student’s *t*-test, *n*=3); and (**m**) survival on PQ exposure, all in the presence and absence of GlcN, respectively (*P*<0.01, log-rank test, *n*=3). The bars represent the mean+s.d. *P<0.05, **P<0.01, ***P<0.001 versus control.

First we have analysed whether GlcN affects growth rates of *C. elegans* food source, alive *Escherichia coli* strain OP50, and found no impact of GlcN on bacterial growth or doubling time (Supplementary Fig. 1a,b). We subsequently determined whether GlcN at a pharmacologically relevant concentration of 100 μM impairs glucose metabolism in wild-type nematodes (*C. elegans* strain Bristol N2), and found glucose oxidation rates to be reduced by 43% (Fig. 1a), indicating that GlcN impairs glucose metabolism, however, to a much lesser extent than DOG does, as previously shown^15^. We then exposed N2 nematodes to the same concentration of GlcN and found their life span to be increased (Fig. 1b) (see Table 1 for life span details, applies to all subsequent *C. elegans* life span analyses; see Supplementary Fig. 1c–e for individual results for data summarized in Fig. 1b). Performing life span assays in a blinded manner produced identical results (Supplementary Fig. 1f). Lower concentrations of GlcN extended life span to a lesser extent (10 μM: mean life span +2.26%,), whereas a higher concentration (1 mM; Tbl. 1) did not extend life span further than the previously determined concentration of 100 μM (Fig. 1b). To further test the possibility that *E. coli*-derived metabolites of GlcN may influence the effects on *C. elegans* longevity, we performed life span assays on heat-inactivated bacteria (Supplementary Fig. 1g) that essentially showed the same results as on alive *E. coli* (Fig. 1b).

### GlcN causes an ATP deficit and promotes mitochondrial biogenesis

We next found that exposure to GlcN for 24 h causes a pronounced decrease in nematodal ATP content (Fig. 1c). A decrease in ATP, that is, available energy, typically activates energy sensors such as AMP-activated protein kinase (AMPK, and its regulatory subunit being known as AAK-2 in nematodes^21^) or, indirectly, specific sirtuins (SIR-2.1 being the key isoform in nematodes). Accordingly, we found increased threonine phosphorylation of AAK-2 following exposure to GlcN (Fig. 1d), indicating activation of a *C. elegans* orthologue of AMPK^21^, while no antibody to detect basal AAK-2 protein expression was available. Consequently, the effect of GlcN on life span was negated in a strain deficient for AAK-2 (Fig. 1e), whereas GlcN still had an effect, albeit reduced, on life span in a strain deficient for SIR-2.1 (Fig. 1f). This indicates that AMPK/AAK-2 activation is required for the life span-extending capabilities of GlcN, whereas SIR-2.1 appears in this regard to be potentially involved, although less essential.

Activation of AMPK is known to promote mitochondrial biogenesis in mammalian tissues^22^. We consistently observed an increase in nematodal content of mitochondrial DNA (mtDNA) (Fig. 1g), reflecting increased mitochondrial mass, that is, increased biogenesis.

### GlcN transiently induces mitochondrial reactive oxygen species formation

AMPK activation typically leads to increased mitochondrial respiration as a consequence of increased mitochondrial biogenesis, thereby reflecting increased metabolism of non-glycolytic substrates, namely fatty acids and amino acids^22^. An increase in mitochondrial respiration following addition of GlcN was consistently observed (Fig. 1h). Reactive oxygen species (ROS) are considered necessary by-products of mitochondrial respiration, and increased respiration causes elevated levels of mitochondrial ROS. We therefore quantified ROS formation using two independent methods and found increases in ROS levels after 48 h of GlcN exposure (Fig. 1i,j), which, notably, is in accordance with findings from *Arabidopsis thaliana* regarding increased ROS levels following GlcN exposure in a hexokinase-dependent manner^23^. However, the ROS levels of *C. elegans* were found to be decreased 7 days after initiation of GlcN exposure (Fig. 1i,j). We found the activities of ROS defence enzymes, specifically superoxide dismutase (Fig. 1k) and catalase (Fig. 1l), to increase 7 days after the addition of GlcN, suggesting that the mitochondrial ROS signal at 48 h (Fig. 1i,j) induces an adaptive response to promote an endogenous defence mechanism alleviating increased ROS levels at 7 days. We next posited whether this increase in ROS defence capacity could possibly contribute to an increased resistance against paraquat (PQ) stress. GlcN-treated worms survived PQ exposure better and longer than their untreated counterparts (Fig. 1m), indicating that GlcN induces a resistance to stress that may contribute to the extension of life span.

### Antioxidants prevent GlcN-mediated life span extension

**Figure 2:**
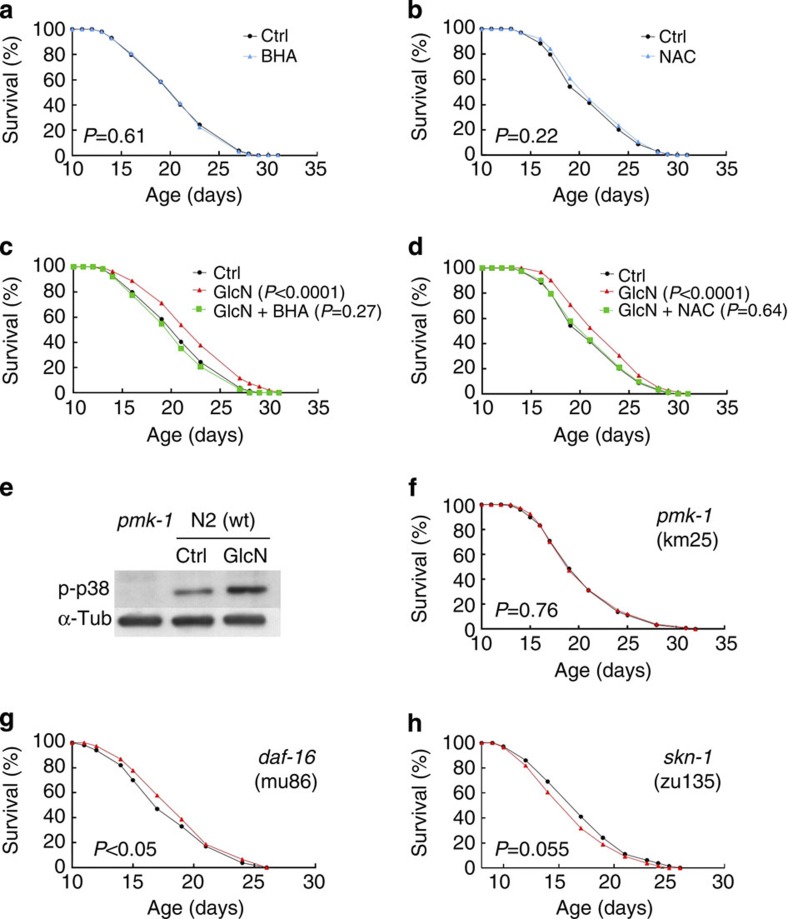
GlcN-induced ROS formation is required for life span extension. Life span assays of N2 wild-type (wt) nematodes in the presence and absence of the antioxidants (**a**) BHA (*P*=0.61, log-rank test, *n*=3) and (**b**) NAC (*P*=0.22, log-rank test, *n*=3). Life span assays of N2 wild-type worms on GlcN (*P*<0.0001, log-rank test, *n*=3), as well as in the co-presence of (**c**) BHA (green; *P*=0.27, log-rank test, *n*=3) and (**d**) NAC (green; *P*=0.64, log-rank test, *n*=3). (**e**) Representative western blot of whole-worm lysates in the presence and absence of GlcN in wild-type worms, as well as untreated PMK-1-deficient worms. Life span assay on (**f**) PMK-1-deficient nematodes in the presence and absence of GlcN (*P*=0.76, log-rank test, *n*=6) on (**g**) DAF-16-deficient nematodes (*P*<0.05, log-rank test, *n*=3) and on (**h**) SKN-1-deficient nematodes (*P*=0.055, log-rank test, *n*=3), all in the presence and absence of GlcN.

Mitochondrial ROS signalling in nematodes^15^,^24^ and, in particular, mitohormesis^15^,^25^ suggests that a low-dose, transient increase in ROS formation promotes metabolic health and life span^26^,^27^,^28^, thereby questioning the free radical theory of ageing^29^. To test whether the increase in ROS (Fig. 1i,j) is essential for a GlcN-mediated extension of life span, we repeated the initial life span experiment (Fig. 1b) in the presence of the antioxidants butylated hydroxyl anisole (BHA) and N-acetyl-cystein (NAC), respectively. Although neither BHA (Fig. 2a) nor NAC (Fig. 2b) had a detectable effect on *C. elegans* life span in the absence of GlcN, the life span-extending capabilities of GlcN were nullified in the presence of BHA or NAC (Fig. 2c,d). This indicates that the transient increase in ROS (Fig. 1i,j) is required for the extension of life span caused by GlcN, thus providing additional support for adaptive ROS signalling^26^,^30^ or mitohormesis^27^,^28^ or both.

### ROS signals are transduced by p38/PMK-1 and NRF-2/SKN-1

Next, we questioned how this essential ROS signal may be sensed and transcriptionally transduced. We initially analysed phosphorylation of a previously established ROS sensor^31^,^32^, p38 MAP kinase, which is called PMK-1 in nematodes, and found increased phosphorylation in GlcN-treated worms (Fig. 2e) while an antibody against basal p38 was not available. GlcN consistently failed to extend the life span of nematodes that are deficient for PMK-1 (Fig. 2f), indicating that the activation of p38 is required to extend life span with GlcN. We then tested whether the absence of downstream transcription factors such as DAF-16 or SKN-1, orthologues of mammalian FoxO and NRF-2, respectively, would influence GlcN effects on life span. We observed a marginally significant effect of GlcN on DAF-16-deficient worms (Fig. 2g), whereas the lack of SKN-1 fully negated the effects of GlcN on life span, even resulting in a shortened life span following GlcN treatment (Fig. 2h). This indicates that SKN-1/NRF-2-dependent initiation of transcription is involved in the GlcN-mediated elongation of *C. elegans* life span.

### GlcN activates identical pathways in worms and mammalian cells

**Figure 3:**
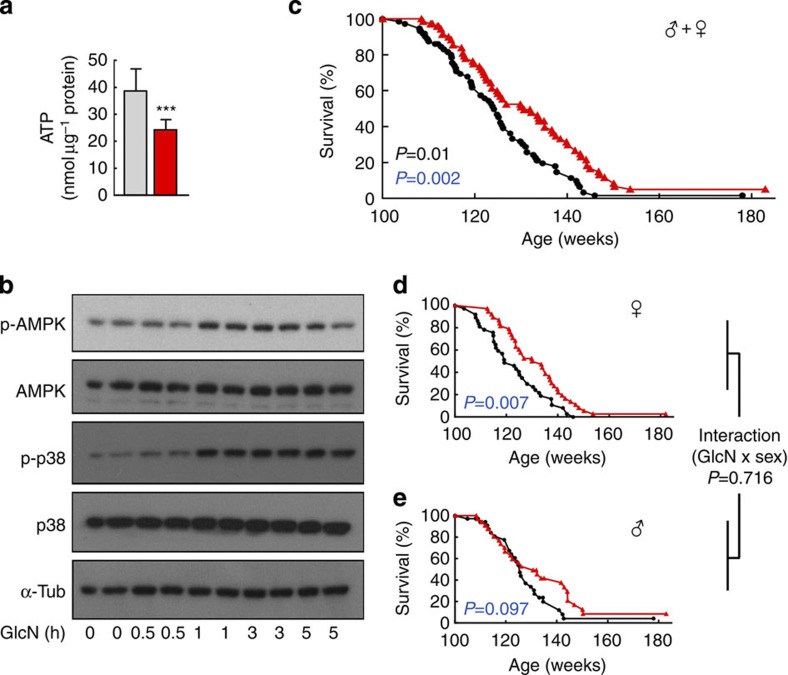
GlcN promotes hepatic energy depletion and increases life span in ageing mice. (**a**) ATP content of HepG2 cells exposed to GlcN for 30 min (bars represent mean+s.d. ***P<0.001 versus unexposed; Student’s *t*-test, *n*=8). (**b**) Representative western blots of HepG2 cells following exposure to GlcN for indicated durations using indicated primary antibodies. (**c**–**e**) Survival curves of C57BL/6NRj mice on a GlcN-containing diet starting at an age of 100 weeks (red) in comparison with control mice. (**c**) Survival of combined male and female C57BL/6NRj mice (log-rank: *P*=0.002; Cox regression: *P*=0.01; *n*=74 control mice and *n*=72 mice on GlcN-containing diet). (**d**) Survival of female C57BL/6NRj mice (log-rank: *P*=0.007; *n*=37 control mice and *n*=38 mice on GlcN-containing diet). (**e**) Survival of male C57BL/6NRj mice (log-rank: *P*=0.097; *n*=37 control mice and *n*=34 mice on GlcN-containing diet); *P*-values that were obtained using Cox regression analyses (including interaction term for ‘treatment by sex’) are given in black font, *P*-values that were calculated using the log-rank test are given in blue font. Controls are always depicted in black or grey colour, whereas GlcN-treatment is depicted in red.

The inhibition of glycolysis by DOG feeding markedly reduces rodent life span^19^ while other glycolytic inhibitors have not yet been tested in this regard. Despite these discouraging findings, we here have tested the effects of GlcN treatment on HepG2 human hepatoma cells and found that GlcN reduces ATP content in such cells (Fig. 3a), which is consistent with GlcN acting as a glycolytic inhibitor^4^,^5^ and reflects the findings in nematodes (Fig. 1c). We next analysed whether pathways contributing to the extension of life span in nematodes could be similarly activated in hepatic cells. We found that GlcN treatment increases Thr172-phosphorylation of AMPK (Fig. 3b) as well as Thr180/Tyr182-phosphorylation of p38 (Fig. 3b) in HepG2 cells, reflecting the findings in nematodes (Figs 1d and 2e, respectively).

### GlcN supplementation extends life span of ageing mice

Based on these promising *ex vivo* observations, we then chronically exposed C57BL/6NRj mice of both sexes, starting at an age of 100 weeks, to GlcN. As the prominent finding of the current study, we observed that GlcN increased the life span of aged mice (Fig. 3c). Both log-rank as well as Cox regression analyses indicated significant differences between controls and GlcN-treated rodents (log-rank: *P*=0.002; Cox regression: *P*=0.01). When applying log-rank statistics to both sexes in separate analyses, it appeared that the response was more pronounced in females (Fig. 3d) than in males (Fig. 3e). Nevertheless, the interaction term for ‘treatment by sex’ within Cox Regression analyses turned out to be insignificant (*P*=0.716), unambiguously indicating that GlcN promotes the life span of both females and males, independent of sex. Calculation of maximum life span^33^ was performed by applying both the Fisher’s exact test and the Z-pooled exact unconditional test. Both tests indicated that maximum life span was extended by GlcN treatment (Fisher’s *P*=0.0143 for 90th percentile, and Z-pooled *P*=0.01255).

### GlcN affects glucose metabolism and mitochondrial biogenesis

**Figure 4:**
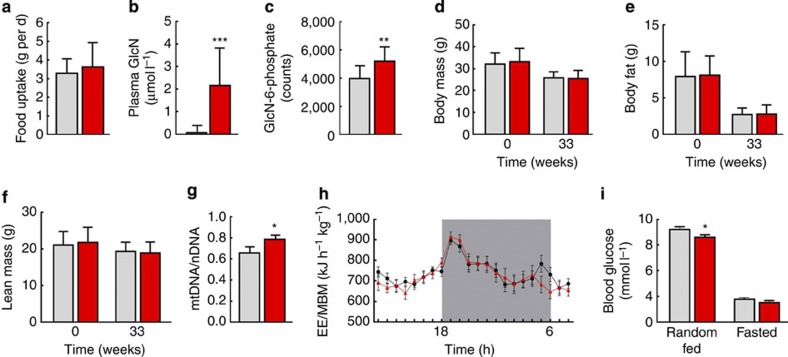
Metabolic consequences of GlcN supplementation. (**a**) Food uptake of C57BL/6-NRj mice (both sexes) chronically exposed to GlcN (red) and respective controls (grey). (**b**) Plasma levels of GlcN in mice (both sexes) on a GlcN-containing diet in comparison with control mice (F(1,33)=27.67, *P*<0.001, *n*=18 control mice and *n*=19 GlcN-fed mice). (**c**) Hepatic levels of GlcN-6-phosphate (F(1,16)=8.74, *P*<0.01, *n*=10 control mice and *n*=10 GlcN-fed mice). (**d**–**f**) Body mass and body composition parameters in such mice. (**g**) Relative mtDNA content in liver specimen (F(1,21)=5.05, *P*<0.05, *n*=12 control mice and *n*=13 GlcN-fed mice). (**h**) Energy expenditure normalized to metabolic body mass of such mice; calculated means for every hour during day, grey area reflects dark phase of the light cycle. (**i**) Random fed (F(1,36)=4.49, *P*<0.05, *n*=20 control mice and *n*=20 GlcN-fed mice) as well as fasting blood glucose levels in such mice. Controls are always depicted in black and grey colour, whereas GlcN-treatment is depicted in red. The bars represent the mean+s.d. *P<0.05, **P<0.01, ***P<0.001 versus control; two-way ANOVA.

We found that food uptake was unaffected not only by the GlcN application but also in regard to the aforementioned interaction term applying two-way analysis of variance (ANOVA) (Fig. 4a and Supplementary Fig. 2a,b). Not surprisingly, GlcN consumption increased blood plasma levels of the compound to the pharmacologically achievable concentrations of ~2 μM (Fig. 4b and Supplementary Fig. 2c,d), as analysed by HPLC. When quantifying the GlcN metabolite GlcN-6-phosphate^4^,^5^, which acts as the competitive inhibitor of glycolysis, by mass spectroscopy, we observed an increase following GlcN consumption (Fig. 4c). The interaction term rejected a sex-specific effect (Supplementary Fig. 2e,f). No differences in murine body mass or body composition were observed before or after treatment with GlcN (Fig. 4d–f and Supplementary Fig. 2g–l).

Treatment of *C. elegans* with GlcN increased nematodal mtDNA content (Fig. 1g). Similarly, we observed an increase in mtDNA content in liver specimen from GlcN-treated mice (Fig. 4g). With the interaction term ‘treatment by sex’ gaining significance (F[1,21]=11.389, *P*=0.003, two-way ANOVA), this effect appears mainly attributable to females (Supplementary Fig. 2m,n). Energy expenditure was then quantified by indirect calorimetry. Although no effect of GlcN feeding on the combined group was detected (Fig. 4h), a trend emerged where female mice appeared to respond to GlcN differently than males (Supplementary Fig. 2o,p).

Chronic GlcN intake has no detectable^11^ or even blood glucose-lowering^12^ effects in humans. We consistently found that GlcN lowered blood glucose in the random fed state (Fig. 4i, left pair of bars), whereas fasting blood glucose levels were not different (Fig. 4i, right pair of bars). No sex-specific effects were observed applying two-way ANOVA statistics for the interaction term ‘treatment by sex’ (Supplementary Fig. 3a,b). Nevertheless, there were no observable differences in glucose tolerance tests (Supplementary Fig. 3c–h). As previously shown, the administration of high-dose GlcN to model systems may cause insulin resistance^6^,^7^,^8^. Accordingly, insulin tolerance tests in GlcN-treated mice revealed a limited degree of insulin resistance at specific time points (Supplementary Fig. 3i–k), whereas the corresponding areas under the curve were different by trend only (Supplementary Fig. 3l–n). There were no observable differences for serum levels of triglycerides (Supplementary Fig. 3o–q), free fatty acids (Supplementary Fig. 3r–t), total cholesterol (Supplementary Fig. 3u–w) or alanine aminotransferase (Supplementary Fig. 3x–z).

### GlcN acts independent of the hexosamine pathway

**Figure 5:**
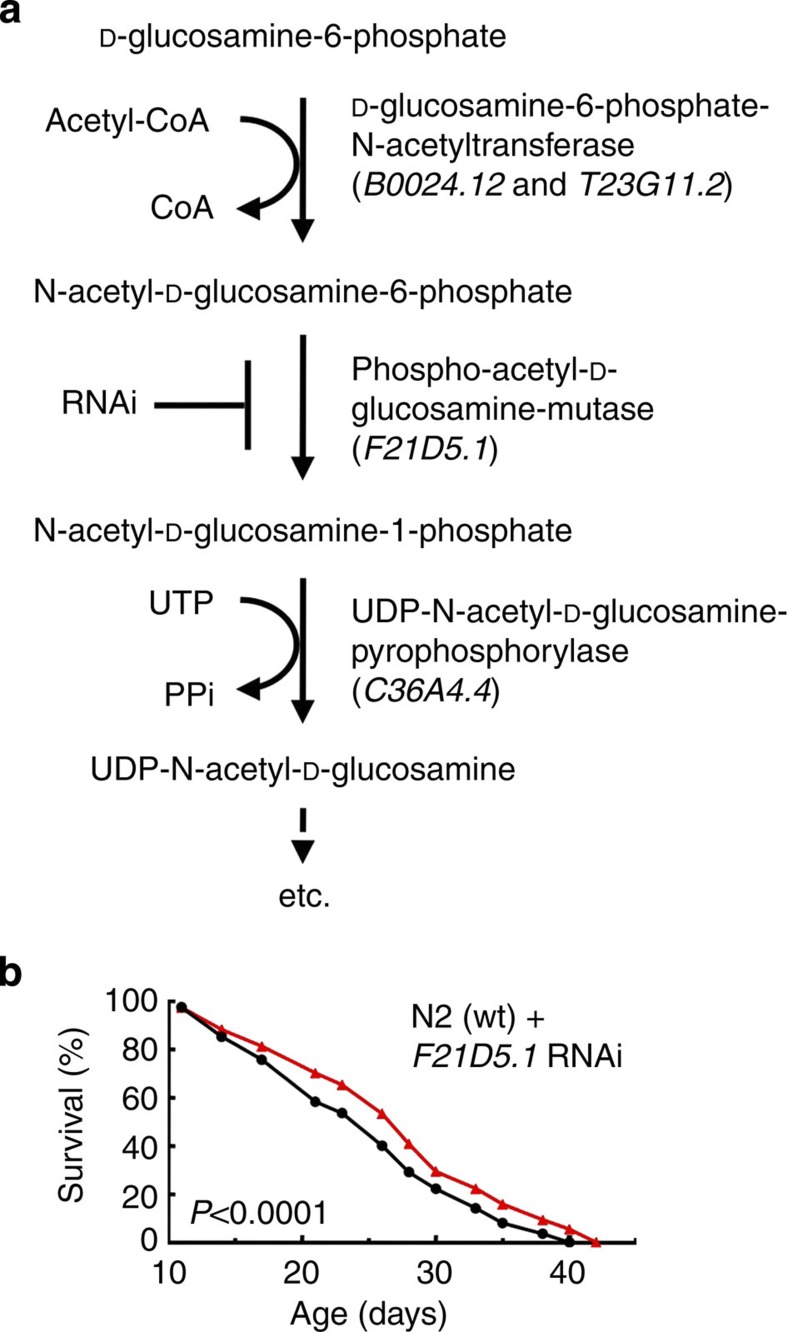
GlcN extends life span independent of the hexosamine pathway. (**a**) Schematic overview on initial enzymatic steps of GlcN metabolism (so-called hexosamine pathway), and the corresponding *C. elegans* orthologues. (**b**) Life span analysis in *C. elegans* treated with RNAi against *F21D5.1* (phospho-acetyl-D-glucosamine-mutase) in the presence (red) and absence of GlcN (*P*<0.0001, log-rank test, *n*=1).

As GlcN-feeding caused increases in GlcN plasma levels (Fig. 4b) and GlcN-6-phosphate concentrations in liver specimen (Fig. 4c), we next tested whether, in addition to inhibiting glycolysis^4^,^5^ (Fig. 1a), metabolites of GlcN-6-phosphate within the hexosamine pathway^34^ (Fig. 5a) contribute to the life span-extending phenotype observed. Unexpectedly, there was no discernable variation in concentrations of the key metabolite UDP-N-acetyl-GlcN (UDP-GlcNAc) (Supplementary Fig. 4a–c) within liver samples, as determined by mass spectroscopy. This insinuates that acetylated metabolites of GlcN-6-phosphate are unlikely to contribute to GlcN-mediated extensions of life span. To further test this, we aimed to disrupt the enzyme GlcN-6-phosphate-N-acetyltransferase (EC 2.3.1.4) (Fig. 5a) that acetylates the GlcN-6-phosphate. Unfortunately, at least two independent genes in worms (Worm Base accession codes *B0024.12* and *T23G11.2*) encode this enzyme^35^ (Fig. 5a), precluding an RNAi-based knockdown approach and appropriate mutants being unavailable. We chose alternatively to impair expression *F21D5.1*, the only *C. elegans* orthologue of mammalian phospho-acetyl-GlcN-mutase (E.C. 5.4.2.3) (Fig. 5a), with RNAi. Such treatment had no effect on the life span-extending capabilities of GlcN treatment in nematodes (Fig. 5b), indicating that inhibition of glycolysis, rather than increased hexosamine metabolism, is responsible for the phenotype observed.

### GlcN induces expression of amino-acid transporters in both species

**Figure 6:**
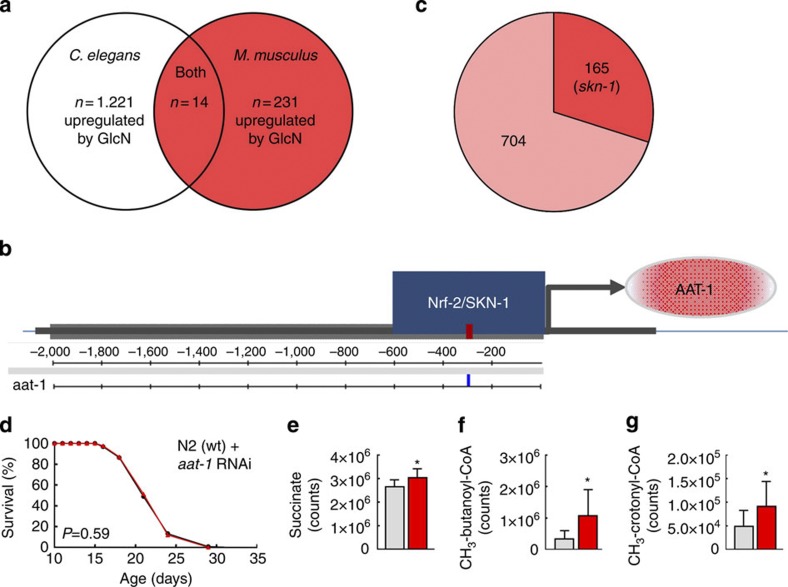
GlcN induces amino-acid uptake and catabolism to extend life span. (**a**) Venn analysis of RNAseq-based mRNA expression levels of genes uniformly upregulated in both *C. elegans* (whole-worm lysates) and *M. musculus* (liver). (**b**) *In silico* promoter analysis of *aat-1* regarding putative binding sites for SKN-1/NRF-2. (**c**) Percentage of SKN-1-dependent genes upregulated following exposure to GlcN in nematodes. (**d**) Life span analysis of nematodes exposed to RNAi against the amino-acid transporter *aat-1* in the presence (red) and absence (black) of GlcN (*P*=0.59, log-rank test, *n*=3). (**e**–**g**) Tissue levels of succinate (F(1,16)=6.46, *P*=0.022, *n*=10 control mice and *n*=10 GlcN-fed mice) (**e**); methyl-butanoyl-CoA (F(1,16)=5.65, *P*=0.03, *n*=10 control mice and *n*=10 GlcN-fed mice) (**f**); and methyl-crotonyl-CoA (F(1,16)=7.97, *P*=0.012, *n*=10 control mice and *n*=10 GlcN-fed mice) (**g**) in liver specimen of mice on a GlcN-containing diet in comparison with control mice. Controls are always depicted in grey colour, whereas GlcN-treatment is depicted in red. The bars represent the mean+s.d. *P<0.05 versus control; two-way ANOVA.

To potentially identify further downstream mechanistic pathways aside from AMPK activation and increased adaptive mtROS signalling via activation of p38 (Figs 1d,e; 2e,f and 3b), we next performed RNA next-generation sequencing analyses on whole-worm extracts as well as liver specimen from mice. In the latter, 231 gene transcripts were found to be induced by long-term exposure to GlcN in comparison with control mice (Fig. 6a and Supplementary Data 1), whereas in *C. elegans*, 1.221 transcripts were found to be upregulated by GlcN (Fig. 6a and Supplementary Data 2). Performing a Venn analysis on these genes for both species, 14 genes were found to be similarly upregulated in both mice and worms (Fig. 6a) based on orthology search (Supplementary Data 3). Out of these, *Slc7a11*, *Slc7a8* and *Slc13a3* (Supplementary Data 1) are amino-acid transporters in mice, whereas *aat-1* is a nematodal amino-acid transporter (Supplementary Data 2), all of which were induced by GlcN in the respective species (Fig. 6a).

We next determined *in silico* analyses of putative promoter elements in the genes upregulated in *C. elegans* (Fig. 6a and Supplementary Data 2). We identified a putative SKN-1-binding site in the promoter of the *aat-1* gene (Fig. 6b) consistent with the life span-shortening effects of GlcN in *skn-1* nematodes (Fig. 2h). Moreover, 36.3% of all GlcN-dependent genes in *C. elegans* carry a putative SKN-1-binding element (Fig. 6c), reiteratively consistent with the experimental data (Fig. 2h).

Then, expression of *aat-1* mRNA in *skn-1* nematodes in the presence and absence of GlcN was determined. Unlike in wild-type worms treated with GlcN deficiency for SKN-1 halted the induction of *aat-1* expression (Supplementary Data 2), insinuating that increased amino-acid uptake mediates the life span-extending effects of GlcN in a SKN-1-dependent manner.

To test this, we impaired *aat-1* expression by RNAi, and observed that the life span-extending effect of GlcN was fully eradicated in these nematodes (Fig. 6d), evidencing that increased amino-acid transport as mediated by AAT-1 is required for GlcN-mediated extension of life span.

### GlcN causes increased catabolism of amino acids

We then determined serum concentrations of urea as a possible indicator for amino-acid turnover in mice, but observed no difference (Supplementary Fig. 4d–f). This result is possibly because of effective excretion of any excess urea via the kidneys.

Finally, we analysed the branched-chain amino acid (BCAA) catabolism^36^,^37^ in liver samples from GlcN-treated mice. Unlike most other amino acids, BCAAs are subject to unidirectional, that is, irreversible catabolism, and individual catabolites can therefore be used to estimate turnover rates. We observed an increase in L-leucine/L-isoleucine catabolites in liver samples of mice (Fig. 6e–g and Supplementary Fig. 4g–l), indicating that increased amino-acid turnover, following chronic GlcN exposure in nematodes (Fig. 6d) and mice (Fig. 6e–g and Supplementary Fig. 4g–l) is linked to life span extension.

## Discussion

The current findings indicate that GlcN at pharmacologically relevant concentrations is capable of extending life span in *C. elegans* and ageing mice. This appears to be a result of decreased glycolysis and a compensatory increase of amino-acid turnover. Although it should be noted that GlcN may impact hexosamine metabolism^34^, we did not, however, detect any differences in downstream metabolites of GlcN-6-phosphate in mice, nor did the disruption of the hexosamine pathway prevent GlcN from extending life span in worms, together strongly suggesting that hexosamine metabolism has no bearing on the phenotypes observed.

Mechanistically, the life span-extending phenotype is rather linked to an activation of SKN-1/NRF-2-dependent transcription, as the latter not only promotes longevity of *C. elegans* independently of GlcN^16^,^32^,^38^,^39^,^40^,^41^,^42^ but also following GlcN exposure (this study). In this regard, it is interesting to note that the *aat-1* gene contains a proximally located SKN-1-binding site and that GlcN exposure induces *aat-1* transcription through SKN-1. This notion is supported by the observations that impairment of either *skn-1* or *aat-1* abrogates the effects of GlcN on life span and disruption of SKN-1 prohibits the induction of *aat-1* expression in nematodes. Interestingly, this upregulation of amino-acid transporters, as well as increased L-leucine/L-isoleucine metabolism, is found in GlcN-exposed liver specimen of rodents, thereby suggesting that the glycolytic inhibitor GlcN induces a metabolic switch towards increased protein metabolism in both worms and mice, unambiguously culminating in extended life span.

Although the specific cause(s) of death of individual mice have not been determined in the current study, it will be interesting to see whether and how GlcN affects not only glucose metabolism, but also cancer growth in chronically supplemented mice, as a number of studies insinuate that GlcN effectively reduces cancer cell proliferation^43^,^44^,^45^,^46^,^47^,^48^.

Unlike for DOG and most other life span-extending compounds, extensive published evidence indicates that GlcN is safe for human use even at high doses, making it readily available for interventions to extend human healthspan particularly because, on an observational and uncontrolled basis, it has been repeatedly suggested that supplementation with GlcN may decrease overall mortality in humans^49^,^50^.

*Note added in proof*: While this publication was in the press, Denzel and co-workers published findings on the role of *N*-acetyl-glucosamine (GlcNAc) supplementation in *C. elegans* lifespan extension, suggesting a different mechanism. This appears to depend on the hexosamine pathway which, however, is unlike to contribute to the phenotype observed in our study (Fig. 5 and Supplementary Fig. 4a–c), indicating that GlcN and GlcNAc promote longevity independently^51^.

## Methods

### Chemicals

All chemicals were obtained from Sigma-Aldrich (Munich, Germany) unless stated otherwise.

### Statistical analyses

Data are expressed as means±s.d. unless otherwise indicated. Statistical analyses for all *C. elegans* data except life span and stress resistance assays were performed by Student’s *t*-test (unpaired, two-tailed) after testing for equal distribution of the data and equal variances within the data set. For comparing significant distributions between different groups in the life span assays and stress resistance assays, statistical calculations were performed using JMP software version 9.0 (SAS Institute Inc., Cary, NC, USA) applying the log-rank test.

Statistical analyses for all murine data except life span analyses were performed by two-way ANOVA including the ‘treatment by sex’ interaction to test for potential sex-specific interactions. In parallel, sex-specific analyses were performed using Student’s *t*-test (unpaired, two-tailed). Mortality rates during the murine survival study were assessed by using log-rank and Cox regression tests to compare survival using SPSS Version 20 (IBM, Armonk, NY, USA). Cox regression was performed including the ‘treatment by sex’ interaction to test for potential sex-specific interactions. Additional calculations were performed using Excel 2007 (Microsoft, Albuquerque, NM, USA). Except for promoter analyses (see below), a *P*-value <0.05 was considered as statistically significant; a *P*-value <0.1 was considered as ‘significant by trend’.

### Nematode strains and maintenance

*C. elegans* strains used for this publication were provided by the *Caenorhabditis* Genetics Center (University of Minnesota, USA). Nematodes were grown and maintained on NGM agar plates at 20 °C using OP50 bacteria as food source^52^. Treatment of *C. elegans* was carried out on NGM agar plates containing 100 μM GlcN, if not stated otherwise. All agar plates were prepared from the same batch of NGM agar, whereas treatment plates were supplemented with the respective compound and control plates with water. After plates were poured and dried, they were sealed and stored at 4 °C. Freshly prepared *E. coli* OP50 were spotted on plates on the previous evening and allowed to dry and settle overnight. Incubations with compounds started 64 h after synchronization of the population, by washing the synchronized, young adult worms and then transferring them to the respective treatment plates using S-Buffer^16^.

### Nematodal life span assays

All life span assays were performed at 20 °C. Sixty-four hours after egg preparation around synchronized 150 nematodes were manually transferred to fresh incubation plates containing the respective compounds. For the first 10–14 days, worms were transferred every day and afterwards every second day. Nematodes that show no reaction to gentle stimulation were scored as dead. Those animals that crawled off the plates or displayed non-natural death particularly because of internal hatching were censored.

### Quantification of *E. coli* growth

Fifty millilitres of liquid Luria Broth (LB) or NGM media were supplemented respectively with either 50 μl H_2_O or 50 μl of a 100 mM GlcN solution. Four Erlenmeyer flasks for each medium and condition were used. Flask were inoculated with 100 μl of an *E. coli* OP50 suspension freshly prepared from an overnight culture. Flasks were put in an orbital shaker (GFL, Burgwedel, Germany) set at 37 °C and 200 r.p.m. Bacterial concentration was determined by measuring optical density at 600 nm in a micro-titer plate reader (FLUOstar Omega, BMG Labtech, Offenburg, Germany).

### RNAi-mediated gene knockdown experiments

For RNAi gene knock-down experiments, we applied *E. coli* HT115 to the worms. Clones for RNAi against *aat-1* and *F21D5.1* were derived from Ahringer library (Source BioScience, Nottingham, UK) and *C. elegans* ORF Collection (Thermo Scientific, Waltham, MA, USA) (Supplementary Table 1) respectively, and were sequenced prior use. The bacteria were spotted on NGM plates containing additionally 1 mM IPTG, 100 μg ml^−1^ Ampicillin and, if required, 12.5 μg ml^−1^ tetracycline (all from Applichem, Darmstadt, Germany).

### [^14^C]Glucose oxidation rates

Uniformly labelled [^14^C]D-glucose was purchased from GE Healthcare. Specific activity of the batch used was 300 mCi mmol^−1^. Animals were maintained as described above on plates containing 5′-fluorouridine and the respective treatment for 24 h. Equal numbers of animals were placed on each plate when treatment was initiated. After collection and three subsequent washes in S-buffer, worm pellets were resuspended in the incubation buffer. Two millilitres of the suspension was transferred to 4 cm Petri dishes. The latter were placed in 10 cm Petri dishes together with a second 4 cm Petri dish containing 600 μl of 0.1 M KOH solution to trap CO_2_^53^. Consequently, each 10 cm dish contained two 4 cm dishes, one carrying nematodes and the other containing KOH. As a substrate, labelled glucose was added to a final concentration of 17.1 μM U-[^14^C]D-glucose (5 μCi ml^−1^) in the nematode suspension. Non-radioactive glucose was added to a final concentration of 0.5 mM to the control samples or the samples pre-treated with GlcN, respectively. The 10 cm Petri dishes were covered, sealed with Parafilm in an air-tight manner and incubated at 20 °C for 60 min. Subsequently, an aliquot of 500 μl of KOH was immersed in 10 ml of scintillation fluid and placed in a liquid scintillation counter (Beckman LS 6000, Global Medical Instrumentation, Inc.) to quantify the amount of trapped ^14^CO_2_. An aliquot was used for protein determination for normalization.

### Respiration assays

Respiration was quantified using a DW1/AD Clark-type electrode (Hansatech, King’s Lynn, England; Great Britain). After the individual incubation period, worms were harvested, washed and transferred into the DW1 chamber. Oxygen consumption was monitored for at least 10 min. Afterwards, worms were carefully removed from the chamber and collected for a subsequent protein determination. Therefore, worms were sonicated three times and centrifuged for 10 min at 12,000 *g*. Supernatant was used for protein determination using BCA, as described below.

### Cell culture

HepG2 hepatoma cells were obtained from LGC Standards (Wesel, Germany) and were grown in RPMI media containing 10% heat-inactivated fetal bovine serum at 37 °C in 5% CO_2_/20% O_2_. Cells were serum starved for 5 h and incubated with or without 5 mM GlcN for the indicated time before collection.

### ATP determination

Worms and cells were harvested and immediately shock frozen in liquid nitrogen. The frozen pellet was grinded in a nitrogen chilled mortar to yield powder. Guanidinium-hydrochloric acid (Guanidium HCl) (4 M) was prepared, heated to 100 °C and then mixed with the frozen powder to destroy ATPase activity and to further lyse samples. The mixture was boiled for 15 min at 100 °C with a subsequent centrifugation step (30 min at 13,200*g* and 4 °C). The supernatant was diluted with ddH_2_O 1:200 and analysed using a commercially available kit (CellTiter Glo; Promega, Fitchburg, WI, USA) according to the manufacturer’s instructions. For normalization of the luminescence signal, protein was determined as described below.

### Immunoblotting

Frozen worm pellets were grinded in a nitrogen-chilled mortar and suspended in phosphate buffer containing protease and phosphatase inhibitors (Complete protease inhibitor cocktail (Roche, Penzberg, Germany) with the addition of 2 mM sodium fluoride, 2 mM sodium ortho-vanadate, 1 mM PMSF and 2 mM EDTA). Cells were harvested with identical buffer. Extracts were sonicated three times and centrifuged for 7 min at 12,000 *g*. Supernatants were used for protein quantification and an aliquot was boiled in Laemmli buffer and applied to SDS-PAGE. Antibodies against the phospho-AMPKα (Thr172; Cell Signaling, no. 2531), basal AMPK (Cell Signaling, no. 2532), phospho-p38 (Thr180/182; Cell Signaling, no. 9211), basal p38 (Cell Signaling, no. 9212) and alpha-tubulin (clone DM1A; Sigma-Aldrich, T9026) were used. Primary antibodies were diluted to 1:1,000 and secondary antibody to 1:10,000 before use on the membranes. Full-length images of immunoblots are shown in Supplementary Fig. 5.

### Protein quantification

Protein content in nematodes and cells was determined by the Bradford method^54^ or the BCA method^55^. Assays were performed in 96-well plates using commercial available kits (Bio-Rad Laboratories AG, Cressier, Switzerland, and Thermo Scientific). Absorbance was measured in a microplate reader (FLUOstar Optima, BMG Labtech, Offenburg, Germany).

### Nematodal quantification of mitochondrial ROS formation

Before ROS measurement MitoTracker Red CM-H_2_X ROS (Invitrogen, Carlsbad, CA, USA) incubation plates were prepared as follows: for each treatment, 500 μl heat-inactivated OP50 (65 °C and 30 min) were mixed with 100 μl MitoTracker Red CM-H_2_X stock solution (100 μM) and spotted on a large NGM agar plate that was allowed to dry for ~20 min^32^. Nematodes were incubated without GlcN, then washed off the plates with S-buffer and then allowed to settle by gravitation to remove offspring. Worms were washed two additional times with S-buffer and centrifuged (300 *g*, 30 s). The worm pellet was transferred to freshly prepared MitoTracker Red CM-H_2_X solution and incubated for 2 h at 20 °C. To remove excess dye from the gut, worms were transferred to NGM agar plates with the corresponding compound or, as a positive control, to plates containing 1 μM rotenone for 1 h at 20 °C. Aliquots of 100 μl worm suspension were distributed into 96-well Fluotrac plates (Greiner Bio-One, Frickenhausen, Germany). Fluorescence intensity was measured in a microplate reader (FLUOstar Optima) using well-scanning mode (excitation wavelength (ex): 570 nm, emission wavelength (em): 610 nm). To normalize fluorescence signal, the remaining worm suspension was used for protein determination.

### Amplex Red-based quantification of supernatant hydrogen peroxide

Worms were removed from plates with 50 mM sodium-phosphate buffer, pH 7.4, washed twice and transferred into an upright Plexiglas cylinder (1.5 ml volume) with continuous stirring at low speed (100 r.p.m.) at 20 °C. First, determination of fluorescence was done without horse radish peroxidase only in the presence of 1 μM Amplex Red (Invitrogen) to detect possible unspecific increase in fluorescence (which was not observed). Next, 0.01 U ml^−1^ horse radish peroxidase was added and changes of fluorescence were recorded with a fluorescence detector (LF402 ProLine, IOM, Berlin, Germany) for at least 15 min at excitation and emission wavelengths of 571 and 585 nm, respectively^42^. Immediately afterwards, worms were removed and collected for protein determination to normalize fluorescence values.

### Superoxide dismutase and catalase activity assays

To determine antioxidant enzyme activities (superoxide dismutase (SOD), catalase (CAT)) nematodes were harvested and washed with ice-cold buffer. The frozen pellet was grinded in a nitrogen chilled mortar together with 200 μl 50 mM phosphate buffer+1 mM EDTA) and sonicated threefold. Lysate was cleared by centrifugation for 15 min at 12,000 *g* and 4 °C. Supernatant was used for the subsequent measurement of catalase or superoxide dismutase activity as well as for a protein quantification to normalize enzyme activities. Determination of catalase activity was performed like previously described with minor changes^56^. Briefly, the diluted (25 mM potassium phosphate buffer plus 1 mM EDTA plus 0.1% BSA, pH 7.5) sample supernatant was mixed with assay buffer (100 mM potassium phosphate buffer, pH 7) and methanol (VWR, Darmstadt, Germany). Hydrogen peroxide (Applichem, 30%) was added and incubated for 20 min under continuous shaking at 20 °C. Reaction was terminated by addition of potassium hydroxide (Applichem, 10 M) and Purpald (Sigma-Aldrich, St. Louis, MO, USA; 46 mM in 0.5 M HCl) and incubated for 10 min by continuous shaking at 20 °C. Potassium periodate (Sigma-Aldrich; 192 mM in 0.5 M potassium hydroxide) was added to oxidize the Purpald–formaldehyde complex and incubated for further 5 min before the absorbance was measured at 540 nm.

SOD activity was quantified using a method described earlier^57^. Sample supernatant was incubated with WST-1 working solution (Tris–HCl, pH 8, diethylene-triamine-penta-acetic acid (Sigma-Aldrich, 100 μM), hypoxanthine (Applichem, 100 μM), WST-1 (180 μM) and xanthine oxidase (Sigma-Aldrich, 240 mU ml^−1^) for 20 min at 37 °C. Then, absorbance was measured at 450 nm.

### PQ stress assay

N2 nematodes at an adult age of 6 days were transferred manually to fresh NGM plates containing 10 mM PQ (Acros Organics, Geel, Belgium) covered with heat-inactivated OP50 (30 min at 65 °C in a water bath) and attended by daily determination of the survival rate until all nematodes were deceased^42^. As described for life span analysis, worms were counted as censored in case of internal hatching, crawling off and bursting.

### Murine breeding and housing conditions

C57BL/6NRj mice were bred in our own facilities based on founders from Janvier Sas (Le Genest Saint Isle, France); it should be noted that these mice lack the *nicotinamide nucleotide transhydrogenase* mutation found in C57BL/6 J-derived strains^58^. Animals were studied starting at an age of 100 weeks. Mice in our colony are tested every 6 months for serologic evidence of viral infection, and all such tests have been negative throughout the period of this study.

The ageing study was initiated with 71 male and 75 female mice in total. These were subjected to GlcN (34 male and 38 female) and control groups (remaining), respectively. Mice were inspected daily for health issues and deaths were recorded for each animal. Moribund animals were killed and recorded.

Mice were housed in a controlled environment (21±1 °C, 12 h/12 h light/dark cycle) and had free access to water and to pellet rodent chow containing 14 mg kg^−1^ alpha-tocopherol and 10 mg kg^−1^ ascorbic acid, as determined by HPLC. At an age of 100 weeks GlcN was supplied at a concentration of 10 g kg^−1^ in the diet (Ssniff Spezialdiäten GmbH, Soest, Germany). All experiments were approved by the Ethics Committee of the State Ministry of Environment, Health and Consumer Protection (Federal States of Brandenburg and Thuringia, Germany).

### Murine body mass and body composition analyses

Body mass was quantified using a graded scale. Body composition was measured by use of quantitative nuclear magnetic resonance technique (Echo MRI-100 Body Composition Analyzer, Echo Medical Systems, Houston, USA) as described^59^.

### Blood sampling

Blood samples, collected in tubes containing 21 U lithium heparin and centrifuged for 10 min at 4 °C and 8,000 r.p.m. (6,800 *g*), were obtained both in the fed state as well as after mice were fasted 16 h overnight by using lancets for submandibular bleeding (Goldenrod Animal Lancet, Medipoint, Mineola, NY, USA).

### GlcN plasma concentrations

Plasma was derivatized^60^ with AccQ-Fluor Reagent Kit (Waters, Milford, MA, USA) according to the manufacturer’s instructions. HPLC was performed on Nexera sytem equipped with degasser DGU-20/A5, auto-sampler SIL-30AC, column oven CTO-20AC and fluorescence detector RF-20AXS (all obtained from Shimadzu, Kyoto, Japan). Chromatographic separation was performed on Reprospher 100 C18-DE 1.8 μm, 50 × 2 mm (Dr Maisch GmbH, Ammerbuch-Entringen, Germany) at 45 °C. Elution was performed using acetonitrile and sodium acetate buffer, pH 5.25, at flow rate of 0.8 ml min^−1^ using a gradient. Detection was carried out using excitation wavelength of 250 nm and emission wavelength of 395 nm.

### Glucose tolerance tests

Glucose tolerance tests were performed by intraperitoneal glucose injection (D-glucose, Merck, Darmstadt, Germany) after mice were fasted 16 h overnight. Plasma was collected before and 10, 30, 60 and 120 min after administering of glucose and immediately frozen at −80 °C for measurement of glucose and insulin^61^.

### Insulin tolerance tests

Mice were subjected to an insulin tolerance test by intraperitoneal injection of 1.5 IU kg^−1^ of human recombinant insulin (Insuman Rapid, Sanofi-Aventis Deutschland GmbH, Frankfurt, Germany). Glucose was determined from a tail vein blood before and 15, 30, 45, 60, 75, and 90 min after insulin injection by a glucometer (Contour, Bayer AG, Leverkusen, Germany).

### Respiratory quotient and total energy expenditure

Total energy expenditure and respiratory quotients were determined by indirect calorimetry at 22 °C for 24 h with an open-circuitry calorimetry system (TSE PhenoMaster System, Inc., Midland, MI, USA). Rates of oxygen consumption (VO_2_) and carbon dioxide production (VCO_2_) were recorded for a 24-h period after mice were allowed to acclimate to the system for a period of 2 days. The air-tight respiratory cages were measured with a flow rate of about 0.38 l min^−1^. VO_2_ and VCO_2_ were recorded for 1.5 min in 16-min intervals for each animal, so that three or four data points were obtained every other hour. TEE (kcal h^−1^) was calculated with the equation TEE=16.17/VO_2_+5.03/VCO_2_−5.98/N, where N is excreted nitrogen and was assumed to be (0.1 g per day). Total energy expenditure was normalized to 24 h and metabolic body mass.

### Determination of plasma parameters

Determination of glucose, triglycerides, alanine-aminotransferase, free fatty acids and total cholesterol in plasma was performed using an automated analyser (Cobas Mira S, Hoffmann-La Roche, Basel, Switzerland) with the appropriate commercially available reagent kits (Glucose HK CP, triglycerides, ALT CP, cholesterol CP, ABX, Montpellier, France; and NEFA HR, Wako, Neuss, Germany).

### Quantification of D-GlcN-6-phosphate

Quantification of GlcN-6-phosphate and its metabolites was performed on an Agilent 6550 QTOF instrument by flow injection analysis time-of-flight mass spectrometry^62^. All samples were injected in duplicates. Ions were annotated based on their accurate mass and the Kyoto Encyclopedia of Genes and Genomes (KEGG) *hsa* reference list allowing a tolerance of 0.001 Da. Unknown ions and those annotated as adducts were discarded. This resulted in a total of 472 putatively annotated ions with unique m/z.

### Targeted analysis of BCAA catabolism

To assess activation of catabolic pathways of BCAAs, we quantified levels of methyl-butanoyl-CoA, methyl-crotonyl-CoA and succinate on a Thermo Quantum Ultra instrument by targeted ion pairing-liquid chromatography tandem mass spectrometry using multiple reaction monitoring^36^,^37^.

### Quantification of mtDNA

Total DNA was isolated by standard proteinase K and phenol–chloroform methods. mtDNA copy number level was analysed by quantitative real-time PCR using Viia 7 (Applied Biosystems). The amount of 0.4 ng of total DNA was used as a template for the amplification of mtDNA in mouse or *C. elegans*. Level of primers against mouse mtDNA (5′-AAGACACCTTGCCTAGCCACAC-3′ and 5′-TGGCTGGCACGAAATTTACC-3′) were normalized against nuclear *18SrRNA* gene (5′-AACTTTCGATGGTAGTCGCCG-3′ and 5′-CCTTGGATGTGGTAGCCGTTT-3′). The same approach was used for analysis of *C. elegans* samples by using primer for mtDNA (5′-CTTTTATTACTCTATATGAGCGTC-3′ and 5′-AACAAAAGAAATTCCTGGTACAAG-3′) normalized against nuclear *18SrRNA* homologue (5′-GCGAAAGCATTTGCCAAGAA-3′ and 5′-ATCGCGAGATGGCATCGTT-3′).

### Extraction of RNA

Total RNA was isolated using QIAzol (Qiagen, Hilden, Germany) based on the phenol–chloroform extraction method. Afterwards, the RNA was quantified photometrically with a NanoDrop 1000 (PeqLab, Erlangen, Germany) and stored at −80 °C until use.

### Next-generation sequencing (RNAseq)

Total RNA was inspected for degradation using Agilent Bioanalyzer 2100 (Agilent Technologies, Santa Clara, CA, USA). For library preparation an amount of 2 μg of total RNA per sample was processed using Illumina’s TruSeq RNA Sample Prep Kit (Illumina; San Diego; CA, USA) following the manufacturer’s instruction. Each library includes its own index sequence to allow multiplexing. The libraries were sequenced using v3 sequencing chemistry and a HiSeq2000 (Illumina) in a single read approach with 50 cycles resulting in reads with a length of 50 nucleotides. Libraries were sequenced in a multiplex manner pooling four libraries per lane. Sequencing ends up with ~30–40 Mio reads per sample. Sequence data were extracted in FastQ format and used for mapping approach.

### Quantification of *aat-1* mRNA

Total RNA was isolated and reverse-transcribed to first-strand cDNA using high-capacity cDNA Reverse Transcription Kit (Applied Biosystems), according to manufacturer’s protocols. Quantitative real-time PCR was performed using optical 384-well plates, SYBR Select Master Mix and Viia 7 (Applied Biosystems). All samples were measured in triplicates, and non-template controls were used to confirm specificity. Expression of *aat-1* (*F27C8.1*) was quantified using specific primers 5′-ACCGGACTTGGTCTCCTTTT-3′ and 5′-TTTGGGTTCTGCAACTCCTC-3′, and normalized against a housekeeping gene (*R07G3.1*) using primers 5′-CTGCTGGACAGGAAGATTACG-3′ and 5′-CTCGGACATTCTCGAATGAAG-3′.

### Bioinformatics of RNA expression data

All reads were mapped against the respective genomic sequences (ce10 for *C. elegans*; mm10 for *Mus musculus*) using TopHat 1.4.1. Only uniquely mappable reads were regarded. For counting the reads per gene (raw counts) the Python package HTSeq ( http://www.huber.embl.de/users/anders/HTSeq/doc/overview.html) was used in mode ‘union’ together with gene annotation for all RefSeq genes downloaded from the UCSC website.

Raw counts for the genes were analysed using the R Statistical Computing Environment^63^ and edgeR^64^. The latter provides statistical routines for determining differential expression in digital gene expression data using a model based on the negative binomial distribution. The resulting *P*-values were adjusted using the Benjamini and Hochberg's approach^65^ for controlling the false discovery rate. If false discovery rate values were <0.05, genes were assigned as differentially expressed. For the comparison of orthologous differentially expressed genes in *C. elegans* and *M. musculus,* the R package orthology was applied^66^.

### Promoter analyses

The search for SKN-1 transcription factor binding sites for each gene was done within the proximal promoter region 2 kb upstream of the predicted start codon. Therefore, a FASTA file containing the promoter regions for all genes was created using WormMart. Next, the remaining sequence file was scanned for one or more matches to the position-specific scoring matrices of SKN-1 using the matrix scan function of the pattern-matching programme regulatory sequence analysis tools^67^. The position-specific scoring matrices contains the nucleotide frequency at each position within the binding sites and were obtained from the Transfac database^68^. The threshold *P*-value, which indicates the risk of false positive predictions, was set to 0.0001.

## Author contributions

S.W., J.P., D.K. and M.R. designed, performed and evaluated all experiments with the following exceptions: K.Z. and J.M. did additional and independent life span experiments in *C. elegans;* K.Z. performed bacterial growth assays; M.G. and M.P. performed next-generation sequencing analysis of mRNA, whereas sample provision, RNA extraction and quality control were done by S.W. and J.P.; bio-informatical evaluation was done by S.P., J.M. and R.G. Promoter analysis was done by J.M. B.L. and K.Z. performed GlcN plasma analysis; S.D. and N.Z. performed all mass spectroscopy data; T.L.M. did all tissue culture experiments; A.F.P. and T.J.S were involved in the study design and contributed several assays; the entire work was supervised by M.R.; the figures were assembled and the manuscript was written by S.W., J.P. and M.R. All authors discussed and commented on the manuscript.

## Additional information

**Accession numbers**: The deep sequencing data have been deposited in NCBI Gene Expression Omnibus under accession code GSE54853.

**How to cite this article:** Weimer, S. *et al.* D-Glucosamine supplementation extends life span of nematodes and of ageing mice. *Nat. Commun.* 5:3563 doi: 10.1038/ncomms4563 (2014).

## Supplementary Material

Supplementary Figures and Table Supplementary Figures 1-5 and Supplementary Table 1

Supplementary Data 1 Differentially expressed genes upon exposure to glucosamine in liver samples of M. musculus

Supplementary Data 3 Genes upregulated in both M. musculus liver specimen and C. elegans lysates upon exposure to glucosamine

## Figures and Tables

**Table 1:** Results and statistical analyses of *C. elegans* life span assays.

**Strain, substance and solvent**	Maximum life span (d) (± s.d.)*	**Mean life span (d) (± s.d.)**	**P-value (versus control, see footnotes)**	**Number of experiments (n)**	**Number of nematodes (n)**
N2 H_2_O (1)	25.00±0.0	22.01		1	135
N2 100 μM GlcN (1)	27.00±0.0	23.82	<0.0001^‡^	1	138
N2 H_2_O (2)	25.00±0.0	21.59		1	135
N2 100 μM GlcN (2)	27.00±0.0	23.67	<0.0001^‡^	1	137
N2 H_2_O (3)	25.00±0.0	21.52		1	150
N2 100 μM GlcN (3)	27.00±0.0	23.14	<0.0001^‡^	1	124
N2 H_2_O (blinded)	24.00±0.0	20.98		1	107
N2 100 μM GlcN (blinded)	26.00±0.0	22.72	<0.0005^‡^	1	126
N2 H_2_O (HIT^†^ bacteria)	28.00	25.52		1	131
N2 100 μM GlcN (HIT^†^ bacteria)	32.00	27.99	<0.0001^§^	1	138
N2 H_2_O	25.00±0.0	21.71±0.3		3	420
N2 10 μM GlcN	25.00±0.0	22.20±0.3	<0.01^‡^	3	428
N2 100 μM GlcN	27.00±0.0	23.54±0.3	<0.0001^‡^	3	399
N2 1 mM GlcN	26.33±1.2	23.33±0.3	<0.0001^‡^	3	442
N2 H_2_O	25.00±0.0	21.50**±**0.6		3	357
N2 100 μM GlcN	27.00±0.0	23.53**±**0.4	<0.0001^‡^	3	408
N2 1 mM GlcN	27.33**±**0.6	23.82**±**0.5	<0.0001^‡^	3	286
N2 10 mM GlcN	27.00±0.0	24.05**±**0.2	<0.0001^‡^	3	286
*aak-2* (ok524) H_2_O	19.67**±**1.2	17.63**±**0.3		3	397
*aak-2* (ok524) GlcN	19.67**±**1.2	17.60**±**0.3	NS^||^	3	397
*sir-2.1* (ok434) H_2_O	22.67**±**0.6	19.50**±**0.4		3	294
*sir-2.1* (ok434) GlcN	23.00±0.0	20.14**±**0.4	<0.05^¶^	3	315
N2 DMSO	23.67**±**1.15	21.06**±**0.42		3	348
N2 GlcN	25.00±0.0	22.53**±**0.8	<0.0001^#^	3	305
N2 BHA	23.00±0.0	20.96**±**0.29	NS^#^	3	320
N2 GlcN/BHA	23.00±0.0	20.67**±**0.16	NS^#^	3	325
N2 H_2_O	24.33**±**1.2	21.38**±**0.3		3	370
N2 GlcN	25.33**±**0.6	22.42**±**0.4	<0.0005^‡^	3	338
N2 NAC	24.67**±**1.5	21.73**±**0.4	NS^‡^	3	372
N2 GlcN/NAC	24.33**±**1.2	21.05**±**0.4	NS^‡^	3	346
*pmk-1* (km25) H_2_O	24±0.0	20.46**±**0.3		6	456
*pmk-1* (km25) GlcN	24±0.0	20.76**±**0.3	NS^**^	6	388
*daf-16* (mu86) H_2_O	21.00±0.0	18.27**±**0.3		3	443
*daf-16* (mu86) GlcN	21.00±0.0	19.01**±**0.3	<0.05^††^	3	431
*skn-1* (zu135) H_2_O	19.67**±**1.2	17.34**±**0.5		3	200
*skn-1* (zu135) GlcN	19.00±0.0	16.64**±**0.5	NS^‡‡^	3	190
*F21D5.1* RNAi/H_2_O	30.0	24.46		1	184
*F21D5.1* RNAi/100 μM GlcN	35.0	28.24	<0.0001^§§^	1	178
*aat-1* RNAi/H_2_O	20.00**±**0.4	21.78**±**0.3		3	214
*aat-1* RNAi/100 μM GlcN	20.00**±**0.4	21.59**±**0.3	NS^||||^	3	225

NS, not significant.*75th percentile; †HIT, heat-inactivated;

‡Controls: N2 H_2_O; ^§^N2 H_2_O HIT bacteria; ||*aak-2* (ok524) H_2_O; ¶*sir-2.1* (ok524) H_2_O; #N2 DMSO; **pmk-1 (km25) H_2_O; ††*daf-16* (mu86) H_2_O; ‡‡*skn-1*(zu135) H_2_O; §§*F21D5.1* RNAi/H_2_O; ||||*aat-1* RNAi/H_2_O.
