# Supplementary material for: D-Glucosamine supplementation extends life span of nematodes and of ageing mice
Source: Nat Commun. 2014 Apr 8;5:3563. doi: 10.1038/ncomms4563 (PMC3988823; doi:10.1038/ncomms4563)
Supplement: Supplementary Figures and Table — Supplementary Figures 1-5 and Supplementary Table 1 [file ncomms4563-s1.pdf]

Supplementary Figure 1

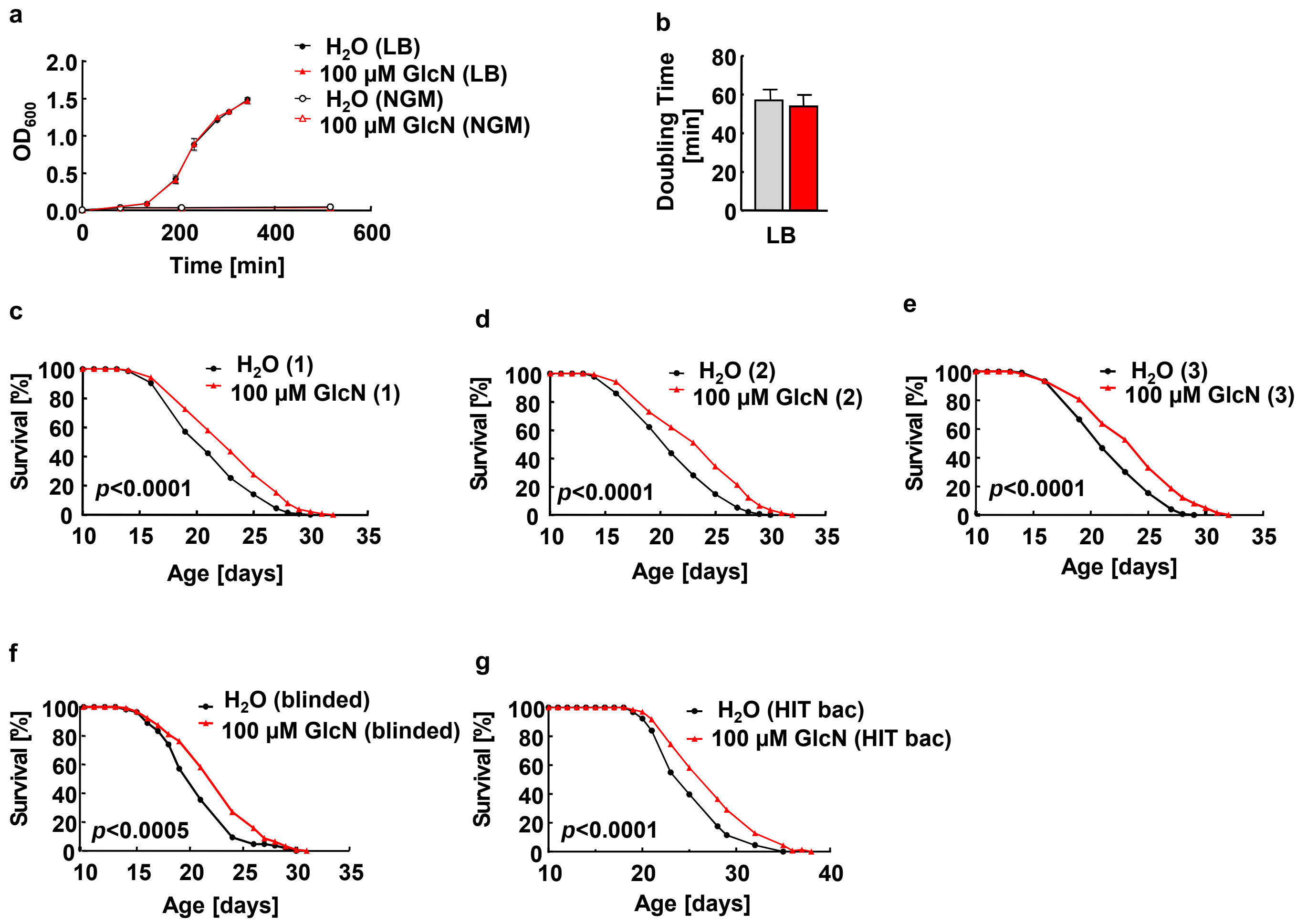

Supplementary Figure 1: Details of C. elegans lifespan assays
Growth characteristics of E. coli OP50 in both LB and NGM media, respectively, depicted **a** as a photometrically determined growth curve and **b** organismal doubling time (for LB media only, since no significant growth in NGM media). **c-e** Individual lifespan results of data summarized in **Fig. 1b** (p<0.0001, log-rank test, n=1 each). **f** Typical lifespan result for experimental setting where experimenter was unaware of GlcN content or control, respectively (p<0.0005, log-rank test, n=1). **g** Lifespan result using heat-inactivated bacteria (p<0.0001, log-rank test, n=1). Controls are always depicted in black and grey color, whereas GlcN-treatment is depicted in red. The bars represent the mean + s.d.

Supplementary Figure 2

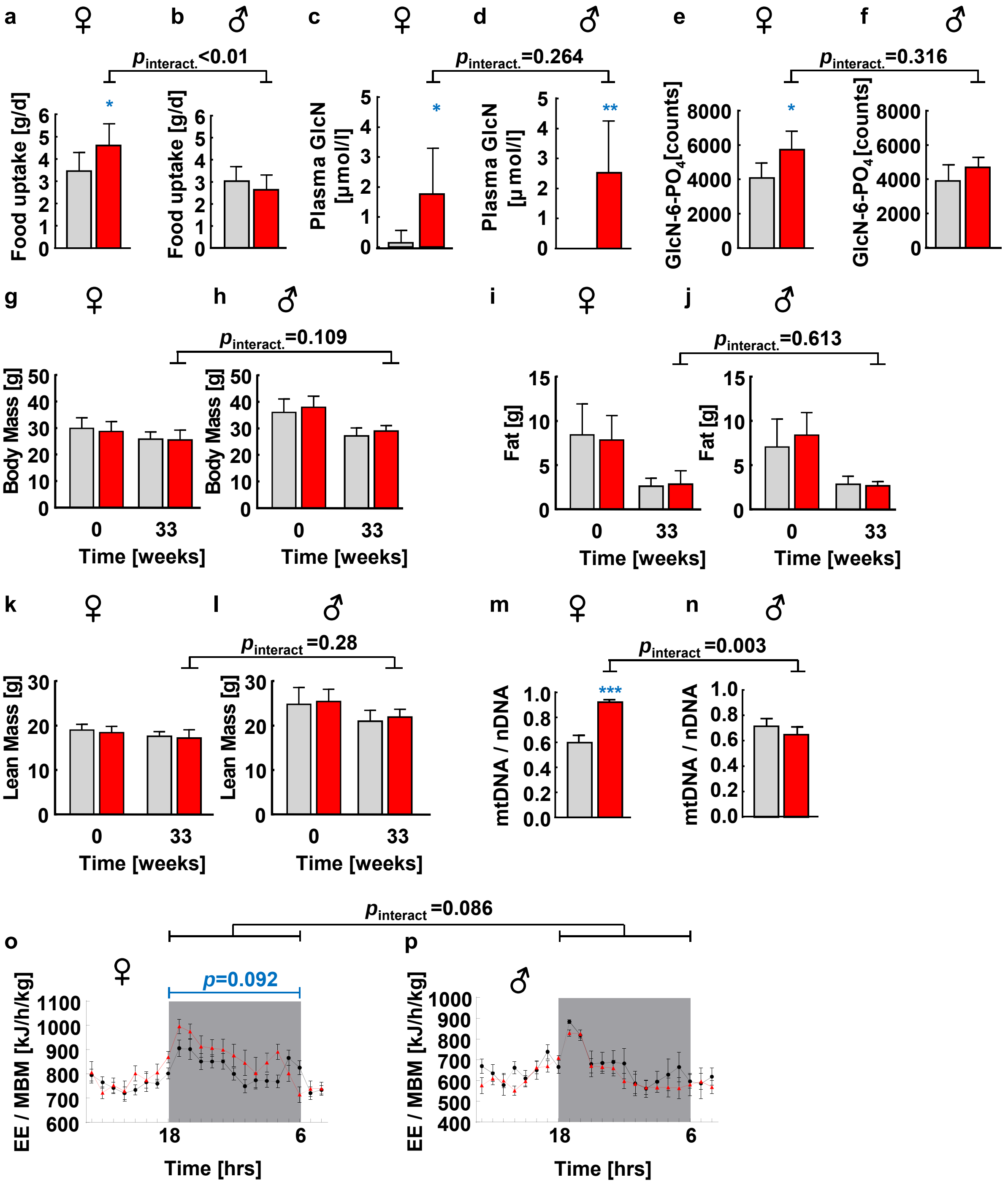

Supplementary Figure 2 continued

Supplementary Figure 2: Sex –specific results of mouse phenotyping part 1
a–b Food uptake of **a** female (blue: $p<0.05$, Student's t-test, n= 10 control mice and n= 10 GlcN-fed mice) and **b** male C57BL/6-NRj mice chronically exposed to GlcN (red), and respective controls (black: interaction between gender and treatment, two-way ANOVA, F (1,33)= 8.22, $p<0.01$, n= 17 control mice and n= 20 GlcN-fed mice). **c** Plasma levels of GlcN in female (blue: $p<0.05$, Student's t-test, n= 10 control mice and n= 9 GlcN-fed mice) and **d** male mice (blue: $p<0.01$, Student's t-test, n= 8 control mice and n= 10 GlcN-fed mice) on a GlcN-containing diet in comparison to control mice (black: interaction between gender and treatment, two-way ANOVA, F (1,33)= 1.29, p=0.264, n= 18 control mice and n= 19 GlcN-fed mice). **e** Hepatic levels of GlcN-6-phosphate in female (blue: $p<0.05$, Student's t-test, n= 5 control mice and n= 5 GlcN-fed mice) and **f** male mice (black: interaction between gender and treatment, two-way ANOVA, F (1,16)= 1.07, p=0.316, n= 10 control mice and n= 10 GlcN-fed mice). **g-l** Body mass (black: interaction between gender and treatment, two-way ANOVA, F (1,34)= 2.71, p=0.109, n= 16 control mice and n= 22 GlcN-fed mice), body fat (black: interaction between gender and treatment, two-way ANOVA, F (1,34)= 0.26, p=0.613, n= 16 control mice and n= 22 GlcN-fed mice) and lean mass (black: interaction between gender and treatment, two-way ANOVA, F (1,34)= 1.21, p=0.28, n= 16 control mice and n= 22 GlcN-fed mice) female and male mice. **m-n** Relative content of mitochondrial DNA in liver specimen of **m** female (blue: $p<0.001$, Student's t-test, n= 6 control mice and n= 5 GlcN-fed mice) and **n** male mice (black: interaction between gender and treatment, two-way ANOVA, F (1,21)= 11.39, p=0.003, n= 12 control mice and n= 13 GlcN-fed mice). **o-p** Energy expenditure normalized to metabolic body mass of **o** female (blue: p=0.092, Student's t-test, n= 10 control mice and n= 10 GlcN-fed mice) and **p** male mice calculated means for every hour during day, grey area reflects dark phase of the light cycle (black: interaction between gender and treatment, two-way ANOVA, F (1,33)= 3.14, p=0.086, n= 17 control mice and n= 20 GlcN-fed mice). Controls are always depicted in black and grey color, whereas GlcN-treatment is depicted in red. The bars represent the mean + s.d.

Supplementary Figure 3

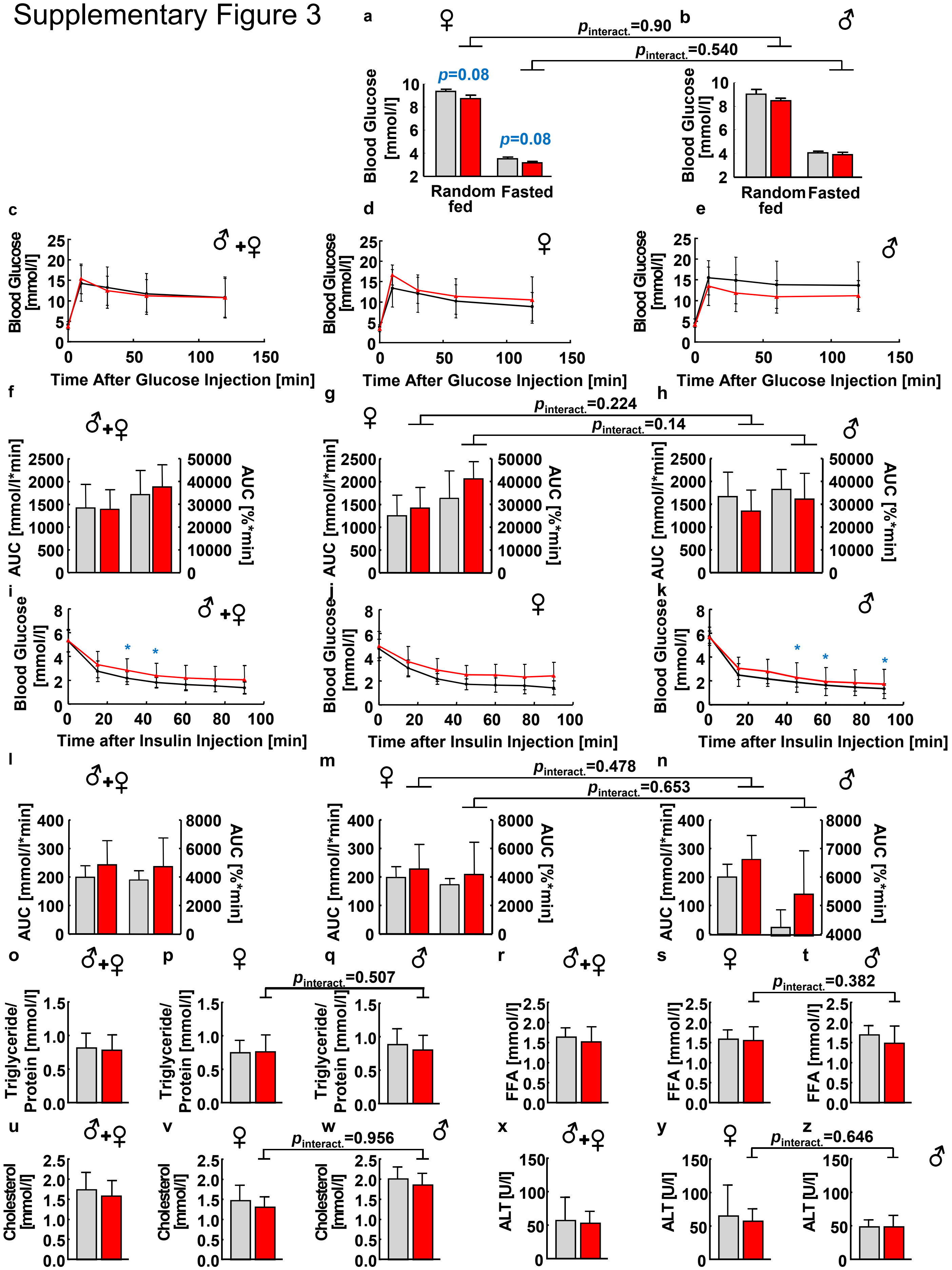

Supplementary Figure 3 continued

Supplementary Figure 3: Sex –specific results of mouse phenotyping part 2
a Blood glucose values of female mice chronically exposed to GlcN (red) in the random fed (blue: *p*=0.08, Student's t-test, n= 10 control mice and n= 10 GlcN-fed mice) and fasted state (blue: *p*=0.08, Student's t-test, n= 10 control mice and n= 10 GlcN-fed mice). **b** Blood glucose values of male mice chronically exposed to GlcN (red) in the random fed and fasted state (black: interaction between gender and treatment, two-way ANOVA, random fed: *F* (1,36)= 0.02, *p*=0.90, n= 20 control mice and n= 20 GlcN-fed mice, fasted: *F* (1,36)= 0.38, *p*=0.540, n= 20 control mice and n= 20 GlcN-fed mice). **c-h** Glucose tolerance tests in mice chronically exposed to GlcN (red). **g** absolute (left y-axis) and relative values (right y-axis) for the area under blood glucose curve (AUC) for female and **h** male mice after chronically exposed to GlcN (red) (interaction between gender and treatment, two-way ANOVA, absolute AUC: *F* (1,23)= 1.56, *p*=0.224, n= 17 control mice and n= 10 GlcN-fed mice, relative AUC: *F* (1,23)= 2.34, *p*=0.14, n= 17 control mice and n= 10 GlcN-fed mice. **i-n** insulin tolerance tests in mice chronically exposed to GlcN (red). **i** Blood glucose at basal level and 15, 30, 45, 60, 75 and 90 min after insulin injection combined for female and male mice (blue: *p*<0.05, Student's t-test, n= 18 control mice and n= 18 GlcN-fed mice). Blood glucose at basal level and 15, 30, 45, 60, 75 and 90 min after insulin injection for **j** female and **k** male (blue: *p*<0.05, Student's t-test, n= 8 control mice and n= 8 GlcN-fed mice). **m** absolute (left y-axis) and relative values (right y-axis) for the area under blood glucose curve (AUC) for female and **n** male mice after chronically exposed to GlcN (red) (interaction between gender and treatment, two-way ANOVA, absolute AUC: *F* (1,32)= 5.12, *p*=0.478, n= 18 control mice and n= 18 GlcN-fed mice, relative AUC: *F* (1,32)= 0.21, *p*=0.653, n= 18 control mice and n= 18 GlcN-fed mice. **o-q** Plasma levels of triglycerides of **p** female and **q** male mice and **o** both sexes in a combined manner chronically exposed to GlcN (red) (interaction between gender and treatment, two-way ANOVA, *F* (1,36)= 0.45, *p*=0.507, n= 20 control mice and n= 20 GlcN-fed mice. **r-t** Plasma level of non-esterified/free fatty acids for both **r** female and male mice combined, as well as for both sexes each (**s** females; **t** males) (interaction between gender and treatment, two-way ANOVA, *F* (1,36)= 0.78, *p*=0.382, n= 20 control mice and n= 20 GlcN-fed mice.) **u-w** Plasma level of total cholesterol for both **u** female and male mice combined, as well as for both sexes each (**v** females; **w** males) (interaction between gender and treatment, two-way ANOVA, *F* (1,36)= 0.003, *p*=0.956, n= 20 control mice and n= 20 GlcN-fed mice.) **x-z** Plasma level of alanine-aminotransferase for both **x** female and male mice combined, as well as for both sexes each (**y** females; **z** males) (interaction between gender and treatment, two-way ANOVA, *F* (1,36)= 0.215, *p*=0.646, n= 20 control mice and n= 20 GlcN-fed mice.) Controls are always depicted in black and grey color, whereas GlcN-treatment is depicted in red. The bars represent the mean + s.d.

Supplementary Figure 4

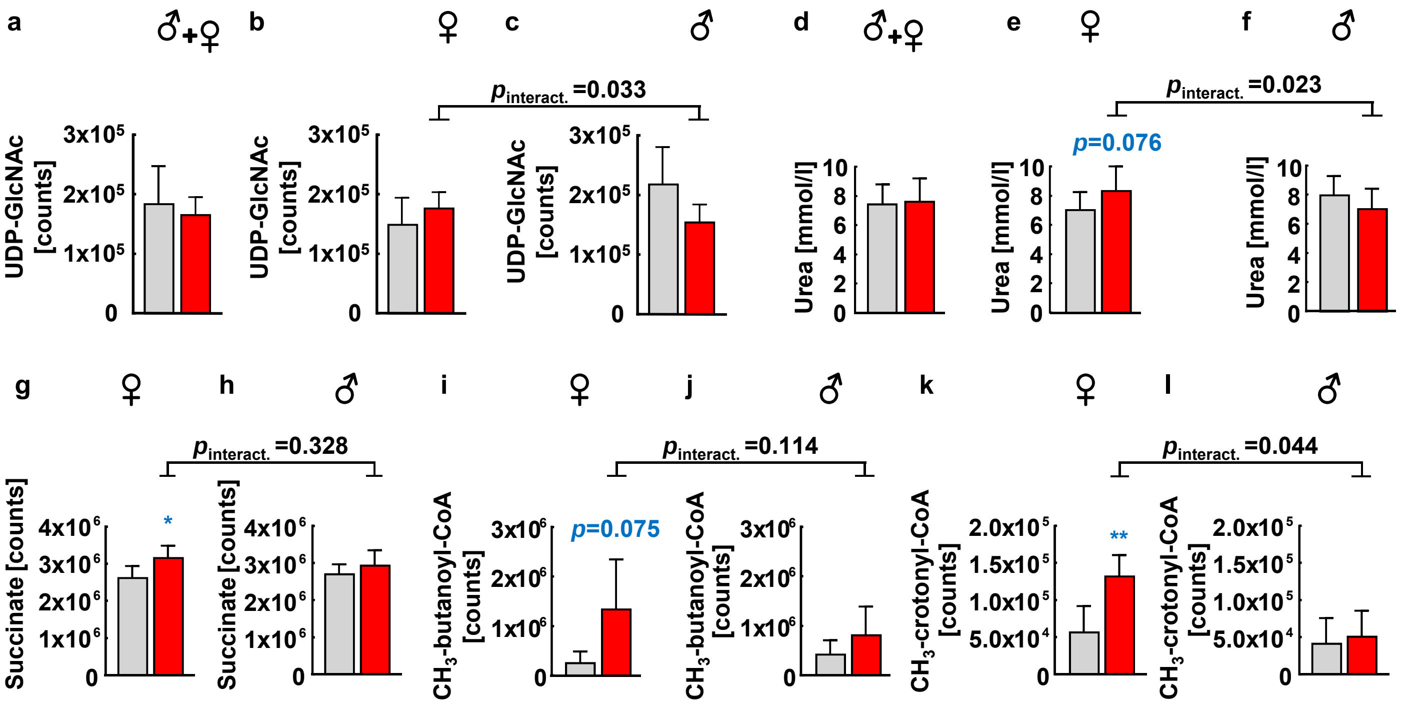

Supplementary Figure 4: Sex –specific results of mouse phenotyping part 3

a-c Concentrations of UDP-N-acetyl-D-glucosamine in liver samples of treated and untreated mice **a** both female and male mice combined, as well as for both sexes each (**b** females; **b** males) (interaction between gender and treatment, two-way ANOVA, F (1,16)= 5.46, p=0.033, n= 20 control mice and n= 20 GlcN-fed mice.) **d** Urea plasma levels of mice chronically exposed to GlcN (red). **e** Urea plasma levels of female mice (blue: p=0.076, Student's t-test, n= 9 control mice and n= 9 GlcN-fed mice) and **f** male mice (interaction between gender and treatment, two-way ANOVA, F (1,32)= 5.68, p=0.023, n= 18 control mice and n= 18 GlcN-fed mice.) **g** liver succinate levels of female (blue: p<0.05, Student's t-test, n= 5 control mice and n= 5 GlcN-fed mice) and **h** male mice (interaction between gender and treatment, two-way ANOVA, F (1,16)= 1.02, p=0.328, n= 10 control mice and n= 10 GlcN-fed mice.) **i** Hepatic levels of methyl-butanoyl-CoA of female (blue: p=0.075, Student's t-test, n= 5 control mice and n= 5 GlcN-fed mice) and **j** male mice (interaction between gender and treatment, two-way ANOVA, F (1,16)= 2.79, p=0.114, n= 10 control mice and n= 10 GlcN-fed mice.) **k** levels of methyl-crotonyl-CoA in liver specimen of control and GlcN-fed female (blue: p<0.01, Student's t-test, n= 5 control mice and n= 5 GlcN-fed mice) and **l** male mice, respectively (interaction between gender and treatment, two-way ANOVA, F (1,16)= 4.78, p=0.044, n= 10 control mice and n= 10 GlcN-fed mice.) . Controls are always depicted in grey color, whereas GlcN-treatment is depicted in red. The bars represent the mean + s.d.

Supplementary Figure 5

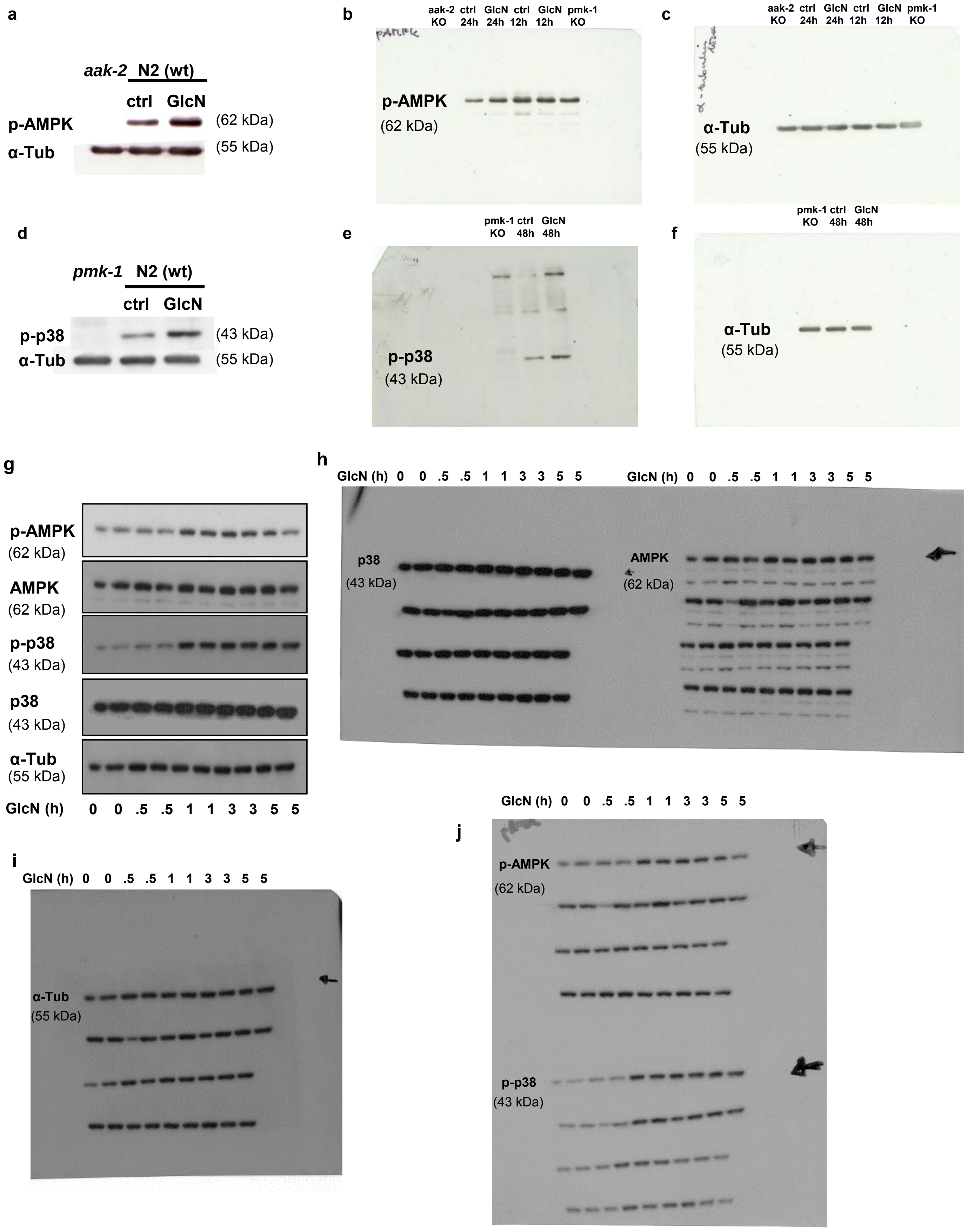

Supplementary Figure 5 continued

Supplementary Figure 5: Membrane scans of Western blots depicted in manuscript main figures

a-f Immunoblotting against phospho-AMPK und phospho-p38 in *aak-2*-KO, *pmk-1*-KO and wildtype worms treated with GlcN and respective controls. Immunoblotting against α-tubulin was used to normalize protein amount. **g-j** Immunoblotting against phospho-AMPK , total AMPK, phospho-p38 and total p38 in HepG2-cells treated with GlcN. Immunoblotting against α-tubulin was used to normalize protein amount. The membranes showing immunoblotting of HepG2 cells were used to detect the signal of several membranes using the same antibody.

Supplementary Table 1

RNAi Sequences

***F21D5.1* cDNA (ORF)**

5’ATGGATCTCACTGTTCCAGTTGAATATTCACGTGCCGGTAACACAACTCACAGCGGTAATGTGCATGCCATTCAAGA
CGATGAGAAATTCTCGTATGGAACTGCTGGATTCCGATTCAAGTCCGAGAAGCTTCCATTCATCGTCTTCCGGTGTGC
TTACGTTGCGAGTCTTCGTGCGCGGCAGCTCAACTCAGCTATCGGAGTGATGATTACAGCTTCACACAATCCATCATG
TGACAATGGTGTGAAATTAGTGGATCCAAGTGGCGATATGCTCAATGAGCAGTGGGAGATATACGCGACTGAAGTTGT
GAATGCTACTGATGCCGAGCTCCCAGCCGCAGTTCGAGCTCTTGAAAAACAAATTTCAGTCGGAAAGACCCAACTTTC
CCGTGTCGTTTGTGGTATGGACACACGTTGCTCTGGTCCTTGTCTGATGAATGCAGCAAGAGCCGGTGCAGCGTTATT
CAATGTACAATTCGATGATATCGGTGTTGTGTCGACTCCAATGCTTCATTATGCTGTCAAGGCATTCAACGAGCCAAAA
TTCGCAGAGCCAACTCACGATGGATATTATTCTGCAATCGCCGATTCGTTCAAGAAGCTGTATGAAATAACTGAGGAAC
CCAAAGACTCGAGATATCAACCAAAAGTCATTGTCGATTGCGCAAACGGAGTCGGTGCTCCACGGTTCAGAAATCTTC
TTGAGCGAATCCCATCATCACTTCTGGAAGTTGAATTTCGTAATGAATCGGAAGAACTGAATCAGGGATGTGGTGCCG
ATTTCGTTAAGATTTCGCAAAAGTTACCAGCAAACTTCTCACCGACAGCAGCAGAACCAAAATGTGCTTCATTTGATGG
AGATGCCGATCGCTTGATGTACTTCCGTGCAAAGGCTTCAGAAAATTCGGAATCAAACGATGCTGAGCTGTTCGACGG
TGACAAAATTGCAGTTCTGATTGTCACATACATTCGGGAGCAACTGAAGGATTACGAAAATTCCACTCCGATGGAACGT
CTCCGCCTTGGTATTGTTCAAACAGCATATGCGAATGGAAGTTCAACACGCTACATTCGTGAAAAGTTGGGTATTGAGC
CAATTATTGTACCTACTGGAGTCAAGCATCTGCATGAAGCTGCTTCTGAGTTCGACATTGGAATTTATTTCGAGGCCAA
CGGACACGGAACTGTTGTTTTCAGCGAGATTTTCGACCGCATCATCAGAAGAACTCCAACTGAATCATTACCTCTCCGT
CGTCTAGCACTTTTCTCCCGTGTCATCAATGAAACTGTTGGGGATGCTTTTGCAGATTTGCTTGCCGTGGAAGCTGTTC
TCCGTCACTATGGATGGTCTATGGATGACTGGGCCGAAAAACTGTATCGAGATGTTCCGAATGTGCAGATCAAAGTTC
CAGTTATTGATCGTTCCATTTTCAAAACGACGAACGCCGAGCAAACTCTTGTGAAACCTGTTGGAATTCAAAAAATGAT
TGATACGGATGTTGCAAAGTACAATAATTCGAGAGCTTTCATCAGACCATCTGGCACCGAGAACATTGTTCGCGTATAC
GCTGAAGCGGATACTGTTGAAAATACATTGCAACTCGGAAAATCTCTCGAACAAGTCGTTCTCAACCTTTGCAACTCGA
ACTGA

***aat-1* (Ahringer library)**

5’CAAGAGCGATACATCCAGCAAGGAAAATAGCTGGCCAAATGAGTGGAACTTTAATTGGTCTTGATGCATCTGGCATG
GTTCTACGAAGCCAGAAGAGTGCGGCAATGGCTGTTCCGATTGCAAGCCAGTAACTGATCTAGAAAATGAAAGTTTCT
TGTTCGAGAACATGCATATTTTTTGAAGCTTGAATTCAAGCTTCTAAAATTGATGCTTCAAGAAACTCAGTACCGGGAAA
TATTAAAAAAAAAAAGCTATCACACTTACTTGAATATAATTAATCAATTGATACACATCCTTTGATGCCAACAAGTAAGCA
ATAGAAAGAGCTCCGGTAAGAATAACAGCGGGAATCGGTGTTTTCGTTTTTTTATTAATCATTGTCAATACAGCTGGCA
TTTGTCCTTCACGAGCACCGGAATAAAAAAGTCTCGCTGAAGTGAAAATAACTCCATTAGCTGATCCAATTGTGGAACA
TGCAACACAGAGTGGCATAATAAATGCGAATTTTCCATAGAGTTTATTGGCGAAAAGTACCGCTACAGCCGGGGATTC
GAGCATTTCATCTGGTGAAATTGCTGTGTAGAGAGCCACATTAGTTAGCACGTAGATTACGGTACAGGATGTGATAGA
GATGGCAATTGCGAGTGGAAGGTTTCTGAAAATTATGGTATAAGAAAATAAATAATATATCGTTAAATTTTTTTTACTGTT
TCATAATGCAATTAGAACCTAAAATAATTTGCTAGATTAATTGACAAGTATTCTACAACAGATTAAAGTAGACCAATAAAC
TACTCATAGTCAGCTCTGAAACTTTAAATAAAAACAATTGGAATGTAAAAACTGTGTTAATGTGTTTCATGTAAACTATAC
AATCACTGAAACTTACCGTTTTGGGTTCTGCAACTCCTCGACGATGAAATTCAAGAAATTCCATCCAGAATAAGCGAAT
AATCCAGAGTAGAAAGCAAGTGAAACTTTTGTAAAATCTTGAGATGTATTCTCAAAAATATTCTCGAATGAGTCCTTGTA
CTGAGATTCACCTGAAATTTTTTATATTTTCTAAATACAGAATAGCTTAAGTGCTCACCAAAGAAAAGGAGACCAAGTCC
GGTTAAAATGATAAGACACAGAGCAACAACCTTTGCAA
